# Metal-Mediated Organocatalysis in Water: Serendipitous Discovery of Aldol Reaction Catalyzed by the [Ru(bpy)_2_(nornicotine)_2_]^2+^ Complex

**DOI:** 10.1021/acs.joc.2c00472

**Published:** 2022-03-25

**Authors:** David Guzmán Ríos, Miguel A. Romero, José A. González-Delgado, Jesús F. Arteaga, Uwe Pischel

**Affiliations:** CIQSO—Center for Research in Sustainable Chemistry and Department of Chemistry, University of Huelva, Campus de El Carmen s/n, Huelva E-21071, Spain

## Abstract

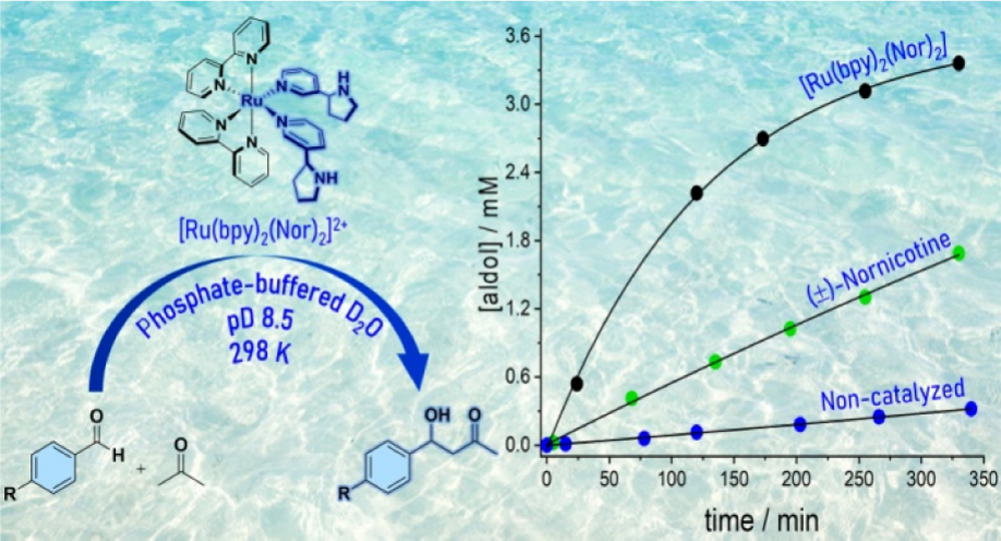

The [Ru(bpy)_2_(Nor)_2_]^2+^ complex (Nor = nornicotine) is an efficient catalyst for the aldol reaction of acetone with activated benzaldehydes in a buffered aqueous solution. The metal plays the role of an activator for the nornicotine organocatalyst ligands. The resulting catalytic activity is potentiated by a factor of about 4.5 as compared to free nornicotine. Similar rate enhancements can be achieved by using Zn(II) cations as the activator. The observations are rationalized with the reduced basicity of the pyrrolidine N in nornicotine due to the enhanced electron withdrawal of the metal-complexed pyridyl moiety.

## Introduction

In the past 2 decades, organocatalysis has developed into an indispensable tool in the synthesis of functional organic molecules, especially those with low molecular weight.^1−7^ A very prominent methodological approach toward organocatalysis is aminocatalysis, implying covalent activation through the formation of enamines or iminium ions between the catalyst and a substrate.^4,6,8,9^ Special focus has been on proline derivatives that can serve as organocatalysts (enamine activation) in aldol reactions,^2,8−11^ being an archetypal carbon–carbon bond formation strategy in synthetic organic chemistry. The efforts dedicated to the development of improved and more efficient catalysts have been always accompanied by concerns regarding the sustainability of organocatalysis. Hence, avoiding or reducing the environmentally hazardous use of organic solvents and shifting this type of chemistry to aqueous media have been continuous and central objectives in the field.^6,8,9,12,13^ In many cases, this challenge was approached by implementing experimental conditions that involve the use of water in the presence of organic solvents.^14−18^ This bears the additional effect that the organic transformations may be accelerated by making use of hydrophobic effects that assist spatial preorganization of the implied reaction partners. One of the first examples for “in-water” enamine catalysis,^19−22^ using a minimum amount of organic co-solvent, was published by the Janda group, who discovered that nornicotine (a nicotine metabolite) works for aldol reactions in buffered aqueous solution.^10,23,24^

**Scheme 1:**
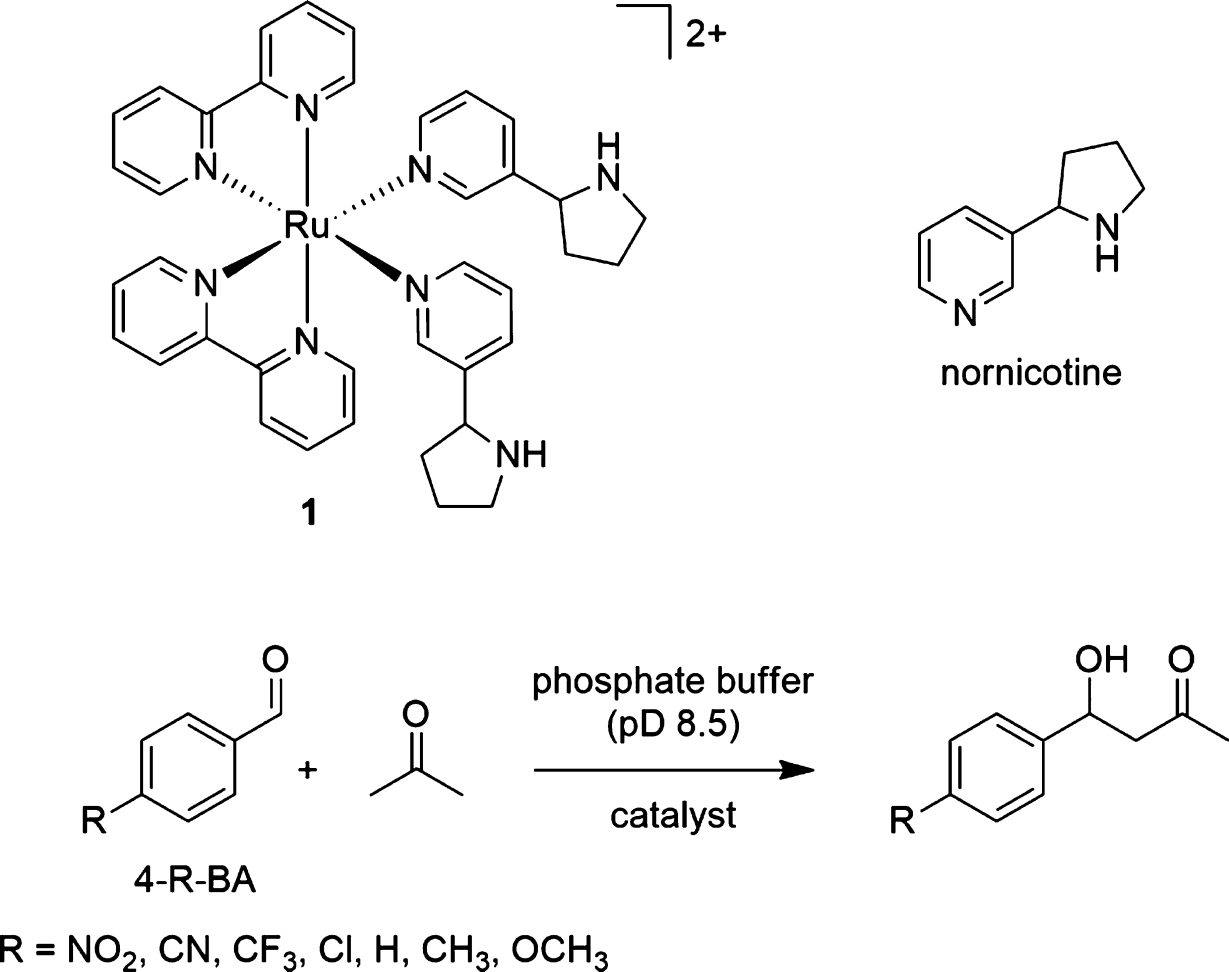
Structures of the Ru(II) Complex **1** and Nornicotine (Employed in Racemic Form, Either as Free Catalyst or Ligand in Complex **1**) Aldol reaction between benzaldehydes and acetone. Note that the aldol is formed as a racemic mixture.

In the quest for photoactivatable organocatalytic systems,^25,26^ we have recently focused on the use of Ru(II)-pyridyl complexes,^27^ which are known to release pyridine ligands on irradiation with visible light.^28^ However, when exploring the complex [Ru(bpy)_2_(Nor)_2_]^2+^ (**1**; Nor = nornicotine; see structure in Scheme 1) with the purpose of photoreleasing nornicotine and initiating a catalyzed aldol reaction between acetone and 4-nitrobenzaldehyde, we noted a significantly accelerated reaction without applying light irradiation. This process was noted to occur even faster than the catalysis by nornicotine alone. Motivated by this observation, we set out to study the process in more detail.

## Results and Discussion

The [Ru(bpy)_2_(Nor)_2_]^2+^ complex (**1**) was prepared from Ru(bpy)_2_Cl_2_ as the precursor and stepwise ligand exchange according to procedures that are reported in the literature.^27,28^ The analytical characterization (^1^H NMR, ^1^H–^1^H COSY, ^13^C NMR DEPT-135, ^13^C NMR DEPTQ-135, ^1^H–^13^C HSQC, high-resolution mass spectrometry, and FT-IR) revealed the identity and purity of the compound; see Figures S1–S7 in the Supporting Information.

**Figure 1:**
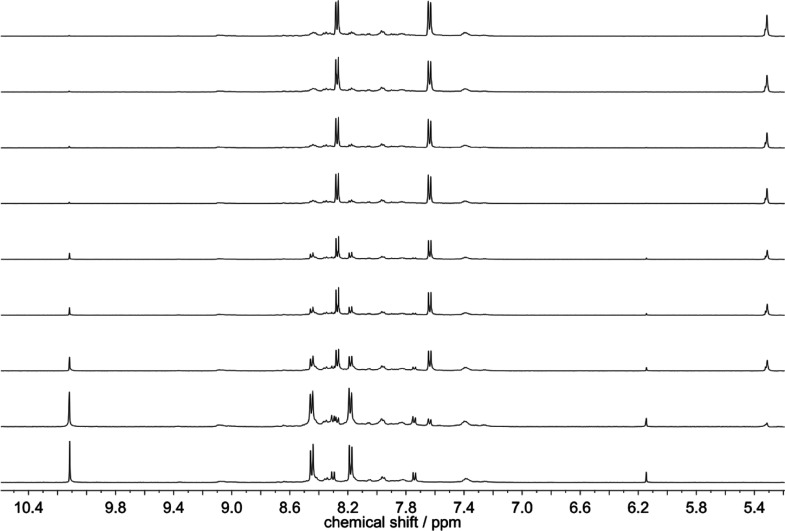
Monitoring of the reaction between 4-NO_2_-BA (3.6 mM) and acetone (270 mM) in the presence of **1** (1.1 mM) in phosphate-buffered D_2_O (45 mM, pD 8.5) at 298 K.

As a starting point, we have chosen the same model reaction as Janda and co-workers in their original work: the aldol reaction between acetone and 4-nitrobenzaldehyde (4-NO_2_-BA) as activated carbonyl compound (see Scheme 1).^10^ The course of the reaction was followed by monitoring characteristic ^1^H NMR signals of the reactant benzaldehyde and the formed aldol product (see Figure 1). 4-NO_2_-BA is accompanied by some amount (*ca.* 20%) of the corresponding hydrate,^29^ being in a chemical equilibrium with the former. The presence of the hydrate is especially evident by the observation of the signal for the CH(OH)_2_ proton at 6.15 ppm. In the course of the aldol reaction, the signals of both forms disappear and the signals of the aldol product are observed. For the benzaldehyde, this is verified for the CHO proton at 10.12 ppm and the aromatic proton signals at 8.18 and 8.45 ppm. On the other hand, the aromatic proton signals at 7.64 and 8.28 ppm and the signal of the CH(OH) proton at 5.31 ppm of the aldol product appear progressively.

**Figure 2:**
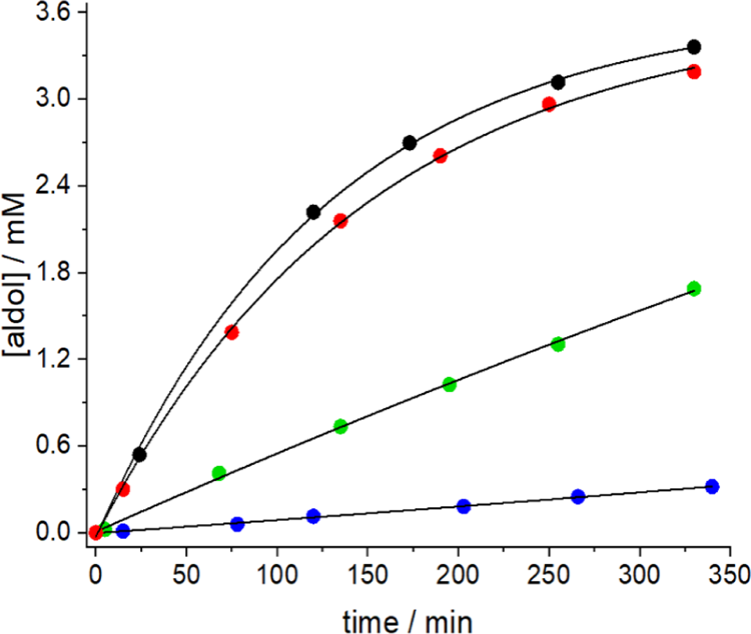
Kinetic curves for the reaction of 4-NO_2_-BA (3.6 mM) with acetone (270 mM) under varying conditions in phosphate-buffered (45 mM, pD 8.5) D_2_O. Black points: presence of **1** (1.1 mM); green points: presence of nornicotine (1.1 mM); blue points: noncatalyzed reaction. The curve constructed from the red points ([4-NO_2_-BA] = 4 mM; [acetone] = 300 mM; [**1**] = 1.2 mM) corresponds to a pre-irradiated sample in 45 mM phosphate-buffered D_2_O (25 min at >455 nm and before adding acetone).

First, we compared the reaction kinetics for the aldol reaction of 270 mM acetone with 3.6 mM 4-NO_2_-BA in phosphate-buffered D_2_O (45 mM, pD 8.5). The corresponding plot for the noncatalyzed reaction and the aldol reaction in the presence of **1** or nornicotine (1.1 mM) is shown in Figure 2. As can be clearly seen, the reaction is considerably accelerated in the presence of **1**, as compared to the nornicotine-catalyzed one or the noncatalyzed background reaction (see corresponding ^1^H NMR monitoring in the Supporting Information—Figures S15 and S16).

Reaction rate constants *k*_obs_ were determined from the slope of a plot of ln([benzaldehyde]) versus time *t*. For all investigated cases (see Table 1), a linear plot was obtained, which is in accordance with a pseudo-first order kinetic regime. The *k*_obs_ values for the noncatalyzed aldol reaction of 4-NO_2_-BA with acetone and the nornicotine-catalyzed version were determined as 3.4 × 10^–4^ and 1.8 × 10^–3^ min^–1^, respectively (see Table 1). These values are *ca*. 2–5 times lower than those reported by the Janda group,^10^ which is explained by the lower temperature at which our study was performed (298 K in this work vs 310 K for the previous study). The *k*_obs_ for the same reaction, but catalyzed by **1**, was measured as 8.5 × 10^–3^ min^–1^. Hence, the presence of **1** yields an additional acceleration of the reaction by a factor of *ca*. 4.7 as compared to free nornicotine.

**Table 1:** Reaction Rate Constants and Chemical Yields for the Aldol Reaction between Acetone and Benzaldehyde Reactants

entry	conditions^a^	*k*_obs_ (min^–1^)^b^	yield (%)^c^
1	**1** (1.1 mM), 4-NO_2_-BA	8.5 × 10^–3^	91
2	**1** (0.55 mM), 4-NO_2_-BA	6.5 × 10^–3^	87
3	**1** (1.2 mM), pre-irradiated >455 nm, 4-NO_2_-BA^d^	7.4 × 10^–3^	88
4	**1** (1.2 mM), pre-irradiated >455 nm, 4-NO_2_-BA, TPPMS (1.2 mM)^d^	7.0 × 10^–3^	86
5	**1** (1.1 mM), 4-NO_2_-BA, TPPMS (9 mM)	7.8 × 10^–3^	90
6	**1** (1.1 mM), 4-NO_2_-BA, TPPMS (26 mM)	5.8 × 10^–3^	83
7	**1** (1.1 mM), 4-NO_2_-BA, TPPMS (50 mM)	1.3 × 10^–3^	32
8	Zn(II) (0.3 mM), (±)-nornicotine (1.2 mM), 4-NO_2_-BA^d^^,^^e^	2.8 × 10^–3^	55
9	Zn(II) (0.6 mM), (±)-nornicotine (1.2 mM), 4-NO_2_-BA^d^^,^^e^	3.4 × 10^–3^	65
10	Zn(II) (1.2 mM), (±)-nornicotine (1.2 mM), 4-NO_2_-BA^d^^,^^e^	4.9 × 10^–3^	78
11	(±)-nornicotine (1.1 mM), 4-NO_2_-BA^f^	1.8 × 10^–3^	43
12	background reaction (non-catalyzed), 4-NO_2_-BA	3.4 × 10^–4^	8
13	**1** (1.1 mM), 4-CN-BA	4.4 × 10^–3^	74
14	**1** (1.1 mM), 4-CF_3_-BA	1.9 × 10^–3^	44
15	**1** (1.1 mM), 4-Cl-BA	5.1 × 10^–4^	13
16	**1** (1.1 mM), 4-H-BA	3.4 × 10^–4^	11
17	**1** (1.1 mM), 4-Me-BA	1.7 × 10^–4^	6
18	**1** (1.1 mM), 4-MeO-BA	4.9 × 10^–5^	2

a The reactions were generally carried out with [4-R-BA] = 3.6 mM and [acetone] = 270 mM in phosphate-buffered D_2_O (45 mM, pD 8.5) at 298 K, except otherwise indicated (entries 3,4, 8, 9, and 10). The structures of the tested benzaldehydes 4-R-BA are given in Scheme 1. TPPMS: triphenylphosphine monosulfonate.

b Pseudo-first-order reaction rate constants, corrected for the minor presence of hydrate for activated benzaldehydes (4-NO_2_-BA, 4-CN-BA, and 4-CF_3_-BA).

c Chemical yield of the aldol product after 5 h, determined by ^1^H NMR spectroscopy.

d [4-NO_2_-BA] = 4 mM, [acetone] = 300 mM.

e Done in a nonbuffered solution (pD 7.4).

f The reaction at pD 7.4 (nonbuffered solution) yields the same reaction rate constant.

Note that the reaction rate constant shows a mild dependence on the catalyst concentration (see Table 1). However, even for 0.55 mM **1** (corresponding effectively to 1.1 mM nornicotine ligands), still a 3.6 times higher *k*_obs_ (6.5 × 10^–3^ min^–1^) was obtained as compared to the reaction that was catalyzed by 1.1 mM free nornicotine. This excludes that a trivial concentration effect is at the origin of the observed acceleration by **1**. Without surprise, the reaction that used **1** as catalyst, translated directly into a higher chemical yield (corresponding to a fixed reaction time of 5 h): 91% for 1.1 mM **1** versus 43% for 1.1 mM nornicotine and merely 8% for the noncatalyzed reaction.

**Figure 3:**
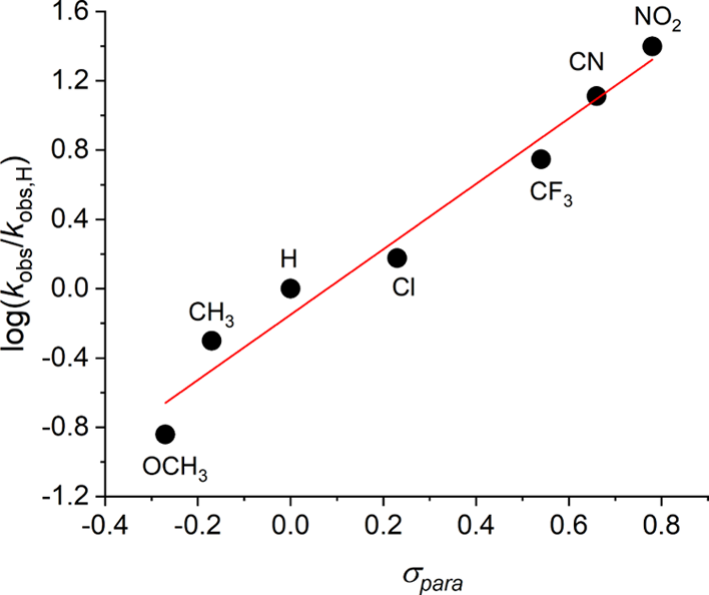
Hammett plot for the aldol reaction between various benzaldehydes and acetone, catalyzed by **1**.

In order to screen the scope of the reaction, we tested several other benzaldehyde-derived reactants (see Scheme 1), varying the electronic demands through the *para* substituent (see rate constants in Table 1 and the Supporting Information for the corresponding ^1^H NMR monitoring—Figures S9, S20–S25). The substituent constants cover an extended range of −0.27 ≤ σ_para_ ≤ +0.78.^30^ The increment of the electrophilic character of the aldehyde carbon by electron accepting substituents is clearly evident, leading to the reactivity order NO_2_ > CN > CF_3_ ≫ Cl > H > CH_3_ > OCH_3_. Even with the less activated 4-CF_3_-BA, the reaction rate constant of the reaction catalyzed by **1** is as high as the nornicotine-catalyzed reaction of the far more activated 4-NO_2_-BA. The corresponding Hammett plot yields a straight line (*n* = 7, *r*^2^ = 0.9695) with a positive slope (see Figure 3) and the reaction constant ρ was determined as +1.89. This relatively large positive value points to the significant susceptibility of the reaction to electron-withdrawal from the reaction center of the benzaldehyde derivative.

The results of the linear-free-energy-relationship analysis are in line with the previously established mechanism of nornicotine-catalyzed aldol reactions between benzaldehydes and acetone.^23^ According to this literature precedence, the C–C bond formation between the enamine and the benzaldehyde reactant is the predominant process of the rate-determining step, which is accompanied by a minor contribution of the posterior C–N bond hydrolysis. Intuitively, the C–C bond formation is favored by an increased electrophilic character of the benzaldehyde carbonyl C atom.

The instrumental role of the Ru(II) metal center in the activation of the nornicotine ligand was confirmed by conducting the experiments (270 mM acetone; 3.6 mM 4-NO_2_-BA; 1.1 mM **1**) in the presence of a strongly Ru(II)-binding phosphine (triphenylphosphine monosulfonate; TPPMS) as a competitive ligand. Increasing concentrations of TPPMS led to a significant slowing down of the aldol reaction (see reaction rate constants in Table 1). For example, for the presence of 50 mM TPPMS, the reaction rate constant was *ca*. 6.5 times smaller than for the absence of the competitor ligand and in absolute terms very close to the rate constant for the reaction catalyzed by free nornicotine. The observations can be interpreted in two scenarios: (a) the competitive displacement of the nornicotine from the ligand sphere of the Ru(II) by TPPMS or (b) the occupation of a previously generated vacancy at the Ru(II) metal center by TPPMS.

**Figure 4:**
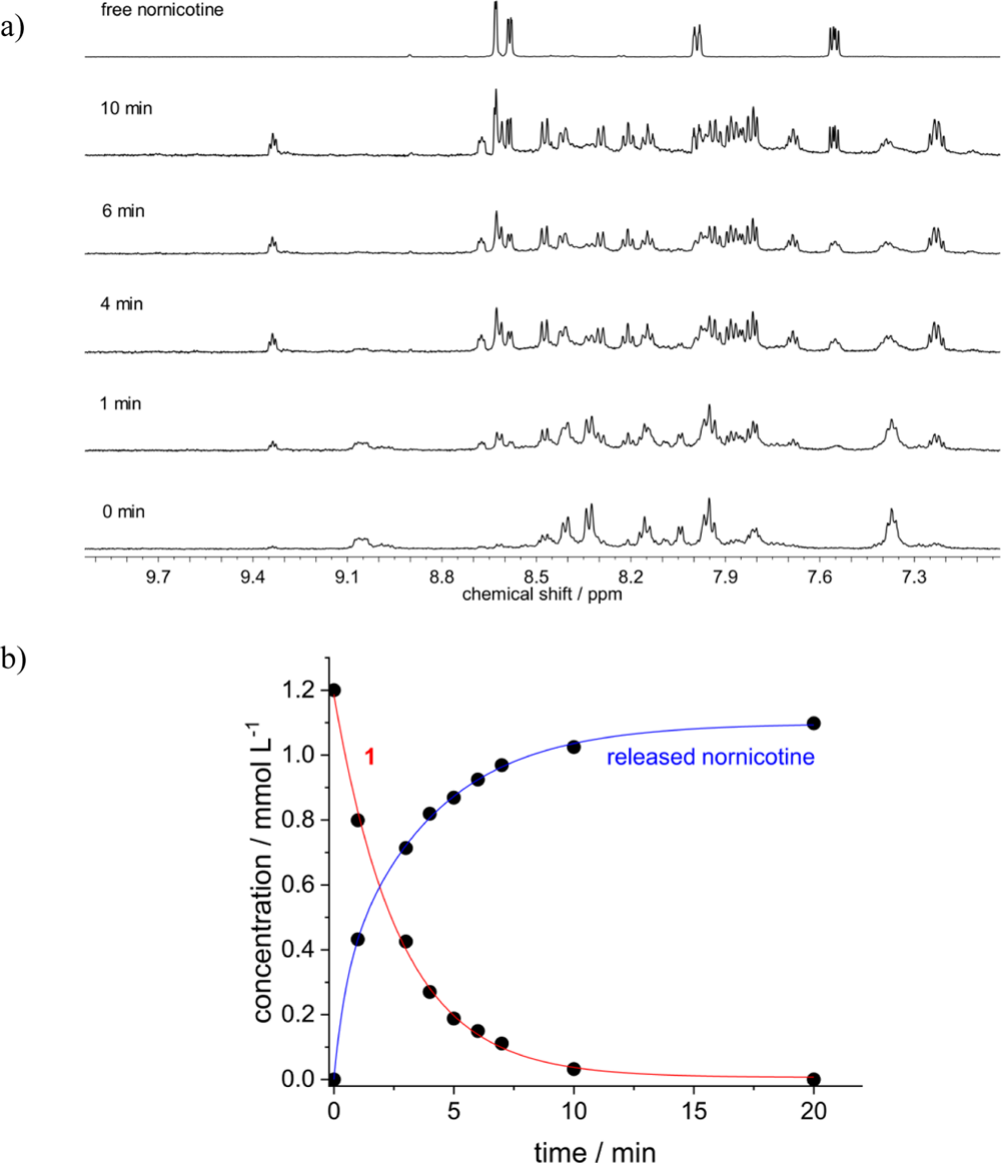
(a) Partial ^1^H NMR spectra, monitoring the photorelease of nornicotine from 1(1.2 mM) in phosphate-buffered D_2_O (50 mM, pD 8.5) at 298 K; irradiation at >455 nm. (b) Corresponding kinetics of the photorelease of nornicotine (blue line) from **1** (red line).

Indeed, as a working hypothesis, it is naturally tempting to postulate the thermal pre-dissociation of one nornicotine ligand, thereby creating a vacancy at the metal center. This could plausibly initiate a catalytic cycle. In this respect, it is important to return for a moment to the starting point of this work, comprising in the exploitation of the photoactivatable release of nornicotine by visible-light irradiation (λ_exc_ > 455 nm, long-pass filter) of **1**. The photoreaction was monitored by ^1^H NMR spectroscopy and UV/vis spectroscopy (see Figure 4 and Figure S27 in the Supporting Information). It leads to the release of one of the nornicotine ligands (photoreaction quantum yield Φ_r_*ca*. 4.5%; determined by ferrioxalate actinometry), as confirmed by the relative integration of ^1^H NMR signals that belong to the released nornicotine and to the remaining complexed ligand. This was corroborated by the mass-spectrometric observation of the corresponding complex, where one of the nornicotine ligands is photosubstituted by water solvent (see Figure S28 in the Supporting Information).

If the hypothesis of creating a vacancy, and thereby initiating a catalytic cycle, would be sustained, then the previous photoactivation of **1**, forming [Ru(bpy)_2_(Nor)(D_2_O)]^2+^, should lead to a faster aldol reaction. A simple comparison of the kinetic curves for the reactions in the dark and for pre-irradiated **1** show that this is not the case. Instead of observing a more efficient reaction for pre-photoactivation, even a slightly less efficient reaction was noted, that is, 8.5 × 10^–3^ s^–1^ for **1** versus 7.4 × 10^–3^ s^–1^ for [Ru(bpy)_2_(Nor)(D_2_O)]^2+^ (see Figure 2 and Figure S13 in the Supporting Information). This is explained by the fact that the photoreleased nornicotine loses the extra activation by metal coordination (see below), with only one nornicotine remaining in the coordination sphere of the Ru center.

In addition, blocking the vacant position at the metal center by competitive displacement of D_2_O in the [Ru(bpy)_2_(Nor)(D_2_O)]^2+^ complex with one equivalent of the much stronger binding TPPMS has no further consequence for the rate constant (*k*_obs_ = 7.0 × 10^–3^ s^–1^). Also, the ^1^H NMR spectrum of **1** (1.2 mM) in phosphate-buffered D_2_O in the presence of acetone (300 mM) does not evidence ligand exchange, as no free nornicotine is observed in the course of 17 h of monitoring. However, mass-spectrometric evidence was obtained for the formation of the nornicotine-derived enamine, with both modified ligands remaining in the coordination sphere of **1** (see Figures S30 and S31 in the Supporting Information). These joint observations support the interpretation that the metal center is not directly involved in the substrate activation, for example, through a type II mechanism, involving enolate stabilization. Instead, the aldol reaction is catalyzed via an enamine mechanism (type I mechanism).^31,32^

Consequently, we turned our attention to coordination-induced changes of the electronic nature of the nornicotine organocatalyst itself. The Janda group reported that the pyridine ring in nornicotine can be replaced with a phenyl ring bearing electronically variable substitution.^24^ They found that strong electron-accepting substituents (such as NO_2_ or CF_3_) further accelerate the aldol reaction between acetone and 4-nitrobenzaldehyde as compared to nornicotine as an organocatalyst. A plausible reasoning for this observation is the reduction of the basicity of the pyrrolidine, leaving a higher percentage of the amine nonprotonated and thus active for the required enamine formation with acetone. The pyridyl moiety in nornicotine has an electron-withdrawing character as compared to a plain phenyl ring. On interaction with positively charged metal cations [such as in the Ru(II) complex **1**] this electron-withdrawing character should be potentiated, leading consequently to further rate enhancement as observed herein.

This result has additional consequences for conducting the nornicotine-catalyzed aldol reaction under physiological conditions. The common presence of biologically relevant metal cations that can coordinate with the pyridine may further accelerate the reaction, similar as observed for complex **1**. To this end, we decided to monitor the reaction kinetics of the aldol reaction between 4-NO_2_-BA and acetone for the presence of Zn(II) cations. Noteworthy, Zn(II) has been used successfully before as Lewis-acid in proline-catalyzed aldol reactions^31−37^ or as a templating agent for the preorganization of bifunctional proline-thiourea organocatalysts.^38,39^

**Figure 5:**
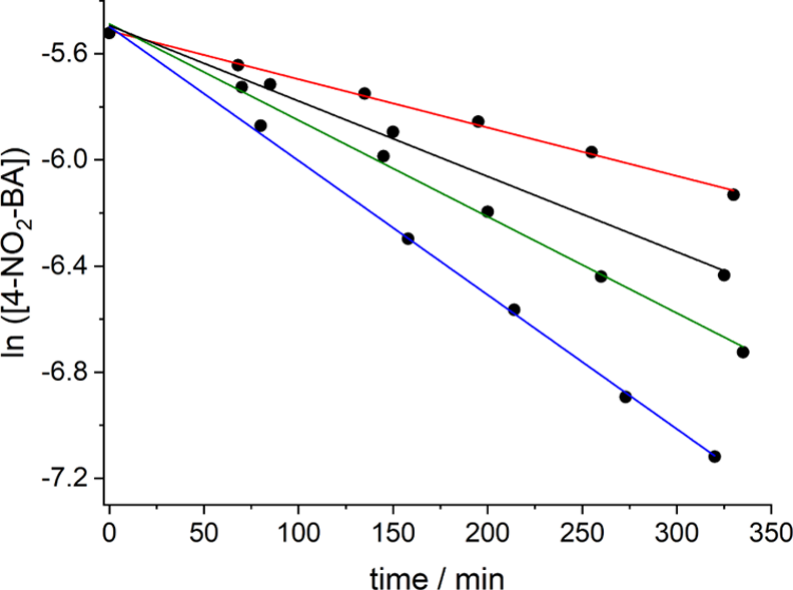
Influence of Zn(II) in the organocatalytic performance of nornicotine (1.2 mM) in the aldol reaction between 4-NO_2_-BA (4 mM) and acetone (300 mM) in nonbuffered D_2_O (pD 7.4); red: absence of Zn(II); black: 0.3 mM Zn(II); green: 0.6 mM Zn(II); and blue: 1.2 mM Zn(II).

In order to avoid deactivation of the Zn(II) in the form of insoluble zinc salt precipitates, the reaction was carried out at pD 7.4 in nonbuffered D_2_O. As shown in Figure 5, increasing amounts of ZnCl_2_ result in a significantly faster reaction, being *ca*. 3 times faster for the presence of 1 equivalent Zn(II) as compared to the sole presence of only nornicotine (4.9 × 10^–3^ min^–1^ vs 1.8 × 10^–3^ min^–1^). Mass spectrometric evidence (see Supporting Information, Figure S29) was obtained for the formation of the [Zn(Nor)_2_]^2+^ complex (Nor = nornicotine). The complex is involved in the formation of the corresponding nornicotine-derived enamine with the ligand remaining attached to the Zn(II) center (see Figure S32). This observation hints on a prevailing type I mechanism (enamine catalysis) and a similar catalyst activation effect by the metal as discussed for the Ru(II) complex. Indeed, the catalysis of the reaction between acetone and 4-nitrobenzaldehyde by Zn(proline)_2_ was shown as well to proceed via the type I mechanism, excluding catalysis by enolate stabilization (type II) mechanism.^31^

## Conclusions

**Scheme 2:**
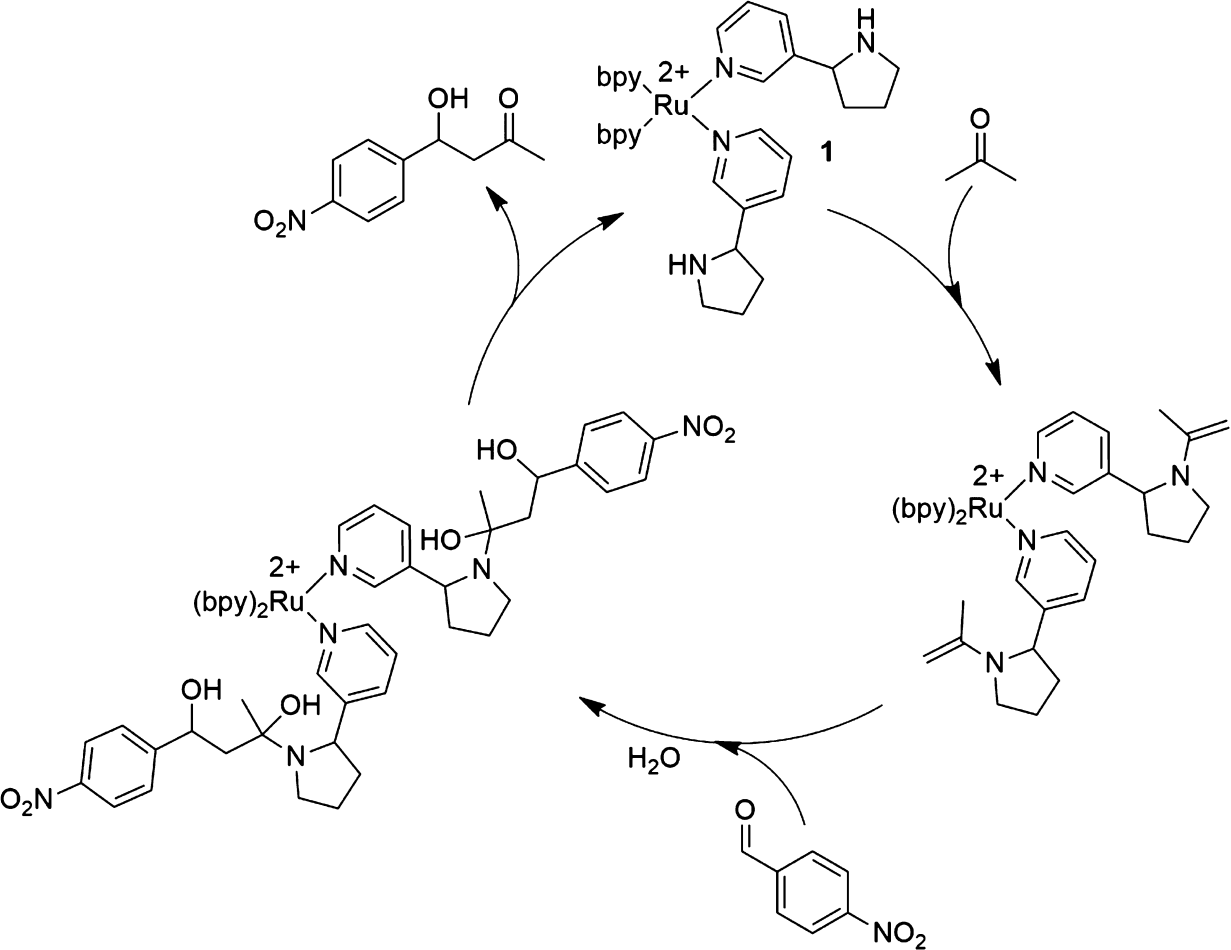
Proposed Catalytic Cycle for the Aldol Reaction of 4-NO_2_-BA with Acetone in the Presence of **1**

In conclusion, we have found that the metal coordination of the pyridyl unit of nornicotine enhances its organocatalytic potential in the aldol reaction of benzaldehydes with acetone (see Scheme 2). The finding of metal-mediated activation of nornicotine provides an additional layer of fine-tuning for this archetypal organocatalyst. The linear free energy relationship analysis points toward an unaltered reaction mechanism with respect to what is established for nornicotine. The role of the metal [Ru(II) in this case] is that of an activator, but it does not participate in the catalytic process itself. This has been further corroborated by making use of the peculiar fact that the [Ru(bpy)_2_(Nor)_2_]^2+^ complex **1** photoreleases one nornicotine ligand. However, the thus created vacancy at the metal center did not lead to a further acceleration of the aldol reaction. The serendipitous finding for the Ru(II) can be expanded toward other metals with more biological relevance. In this context, we found that the nornicotine catalytic efficiency is also enhanced by the presence of Zn(II) ions.

## Experimental Section

### General

All reagents were obtained from commercial sources and used without further purification unless otherwise indicated. Ru(bpy)_2_Cl_2_, the benzaldehyde derivatives (4-R-BA, R = NO_2_, CN, CF_3_, Cl, H, CH_3_, and OCH_3_), acetone, ZnCl_2_, 3-(diphenylphosphino) benzenesulfonic acid sodium salt (triphenylphosphine monosulfonate; TPPMS), and ammonium hexafluoro phosphate (NH_4_PF_6_) were purchased from Sigma-Aldrich. (±)-(Pyrrolidin-2-yl)pyridine [(±)-nornicotine] was from Apollo Scientific. The NMR spectra were recorded on a 500 MHz spectrometer (Bruker 500 MR). Methanol-*d*_4_, dimethylsulfoxide-*d*_6_, and D_2_O (all 99 atom-% D) were used as solvents, and the spectra were referenced to the residual solvent peak (3.31, 2.50, and 4.79 ppm, respectively). The pD was adjusted with DCl or NaOD. Corrections due to isotope effects were applied using the equation pD = pH* + 0.4, where pH* is the reading taken from the pH meter.^40^ Photoirradiation experiments were conducted with a 150 W Xe lamp (LOT ORIEL) using a 455 nm long-pass filter. The irradiations were done with solutions contained in a 1-cm quartz cuvette or in an NMR tube at a 40 cm distance from the light source. Fourier transform infrared (FTIR) spectroscopy measurements were performed using a FT/IR 4200 spectrometer (Jasco, Tokyo, Japan). High-resolution mass spectrometry (HRMS) was performed using an Elite QTOF, Bruker Daltonics autoflex MALDI-TOF. UV/vis absorption spectra were recorded using a Shimadzu UV-1603 spectrophotometer. Structural assignments were made with additional information from gCOSY and gHSQC experiments.

### Synthesis of *cis*-Di[(±)-3-(pyrrolidin-2-yl)pyridine)](2,2′-bipyridine)ruthenium(II) Dichloride (**1**)

A suspension of Ru(bpy)_2_Cl_2_ (159 mg, 0.33 mmol) in water (7 mL), previously deoxygenated by bubbling with nitrogen gas during 15 min, was heated at 358 K until complete dissolution. Then, (±)-3-(pyrrolidin-2-yl)pyridine [(±)-nornicotine, 102 mg, 0.69 mmol, 0.1 mL] was added and the solution was heated at 358 K during 48 h. The compound was precipitated by the addition of NH_4_PF_6_, washed, and dried. The orange-red crude product of oily consistency was diluted in acetone and filtered through DowexS 1X8 (chloride form). The solvent was removed under reduced pressure, obtaining a dark orange solid (208 mg, yield 81%). ^1^H NMR (500 MHz, CD_3_OD): δ 9.10 (dd, *J* = 9.4, 5.1 Hz, 2H), 8.55 (d, *J* = 8.0 Hz, 3H), 8.48 (d, *J* = 7.9 Hz, 3H), 8.38 (d, *J* = 5.6 Hz, 2H), 8.21 (t, *J* = 7.9 Hz, 2H), 8.06 (d, *J* = 5.8 Hz, 2H), 8.00 (t, *J* = 7.9 Hz, 3H), 7.90 (m, 5H), 7.48 (m, 2H), 7.37 (dd, *J* = 9.9, 3.8 Hz, 2H), 4.17 (m, 2H), 3.04 (m, 4H), 2.15 (m, 2H), 1.85 (m, 4H), 1.50 (m, 2H) ppm. ^13^C{^1^H} NMR (126 MHz, CD_3_OD): δ 159.3, 158.9, 154.2, 154.1, 153.9, 153.8, 153.7, 153.5, 153.4, 139.4, 139.1, 137.9, 137.7, 129.7, 129.1, 127.4, 127.3, 125.2, 125.0, 60.8, 60.7, 47.6, 47.5, 34.7, 34.4, 34.3, 26.0 ppm. HRMS (ESI): *m*/*z* calcd for C_38_H_40_N_8_Ru, [M^2+^ – 2Cl] 355.1192; found, 355.1205. FTIR (nujol) *ν*_max_ 3442, 2924, 2854, 2726, 1639, 1461, 1377, 1305, 845, 722 cm^–1^. UV/vis (20 μM in phosphate-buffered D_2_O) λ_max_ (log ε/M^–1^cm^–1^): 245 (4.68), 298 (5.00), 345 (4.37), 452 (4.20) nm.

### Rate Constant Determination

The rate constant for each substrate was determined by the method of initial rates under pseudo-first-order conditions. The assay was realized by preparing a solution of the catalyst [**1** or (±)-nornicotine] in phosphate-buffered D_2_O (pD 8.5) and adding the benzaldehyde 4-R-BA in the form of a stock solution (50 mM) in dimethylsulfoxide-*d*_6_. This constituted the zero time solution. To this mixture, acetone was added in order to initiate the reaction. The amount of dimethylsulfoxide-*d*_6_ co-solvent in the aqueous solution was 7 vol %. For the presence of Zn(II) cations, the reaction was carried out in nonbuffered D_2_O and the pD was lowered to 7.4 in order to avoid the precipitation of insoluble zinc salts, such as hydroxides. The reaction kinetics was followed by ^1^H NMR spectroscopy. The treatment of the kinetic data for the activated benzaldehydes 4-NO_2_-BA, 4-CN-BA, and 4-CF_3_-BA takes the small amount of hydrate into account.

## Supporting Information Available

NMR spectra, FT-IR spectrum, HRMS, and UV/vis absorption spectrum of **1**; NMR monitoring of aldol reactions with benzaldehydes; and photorelease of nornicotine (PDF)
