# Supplementary material for: Metal-Mediated Organocatalysis in Water: Serendipitous Discovery of Aldol Reaction Catalyzed by the [Ru(bpy)2(nornicotine)2]2+ Complex
Source: J Org Chem. 2022 Mar 25;87(8):5412–8. doi: 10.1021/acs.joc.2c00472 (PMC10550203; doi:10.1021/acs.joc.2c00472)
Supplement: Supplementary file 1 — jo2c00472_si_001.pdf [file jo2c00472_si_001.pdf]

Supporting Information (30 pages)

Metal-Mediated Organocatalysis in Water: Serendipitous Discovery of Aldol Reaction Catalyzed by the $[Ru(bpy)_2(nornicotine)_2]^{2+}$ Complex

David Guzmán Ríos, Miguel A. Romero, José A. González-Delgado, Jesús F. Arteaga,* Uwe Pischel*

CIQSO – Center for Research in Sustainable Chemistry and Department of Chemistry, University of Huelva, Campus de El Carmen s/n, E-21071 Huelva, Spain

** Corresponding authors: jesus.fernandez@diq.uhu.es (J.F.A.), uwe.pischel@diq.uhu.es (U.P.)*

Table of Contents

1. Characterization of catalyst **1** by NMR spectroscopy	S2
2. Characterization of catalyst **1** by HRMS, UV/vis, and FTIR	S5
3. Kinetic assays monitored by ^{1}H NMR spectroscopy	S7
4. Photorelease of nornicotine monitored by UV/vis absorption spectroscopy	S25
5. High-resolution mass spectrometry of the Zn(II) complex with nornicotine	S27
6. High-resolution mass spectrometric evidence for enamine formation	S28

1. Characterization of catalyst 1 by NMR spectroscopy

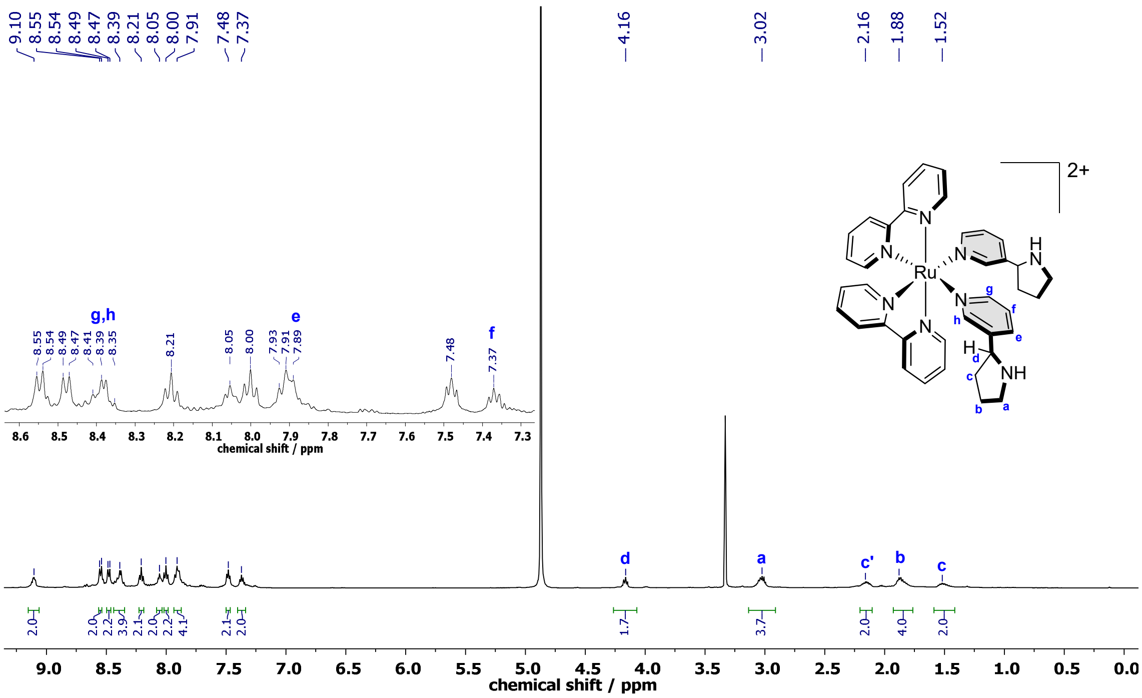

Figure S1. ^{1}H NMR spectrum (500 MHz) of **1** in CD_3OD at 298 K. The inset shows the aromatic region between 7.3–8.7 ppm and the proton assignment of the nornicotine ligand.

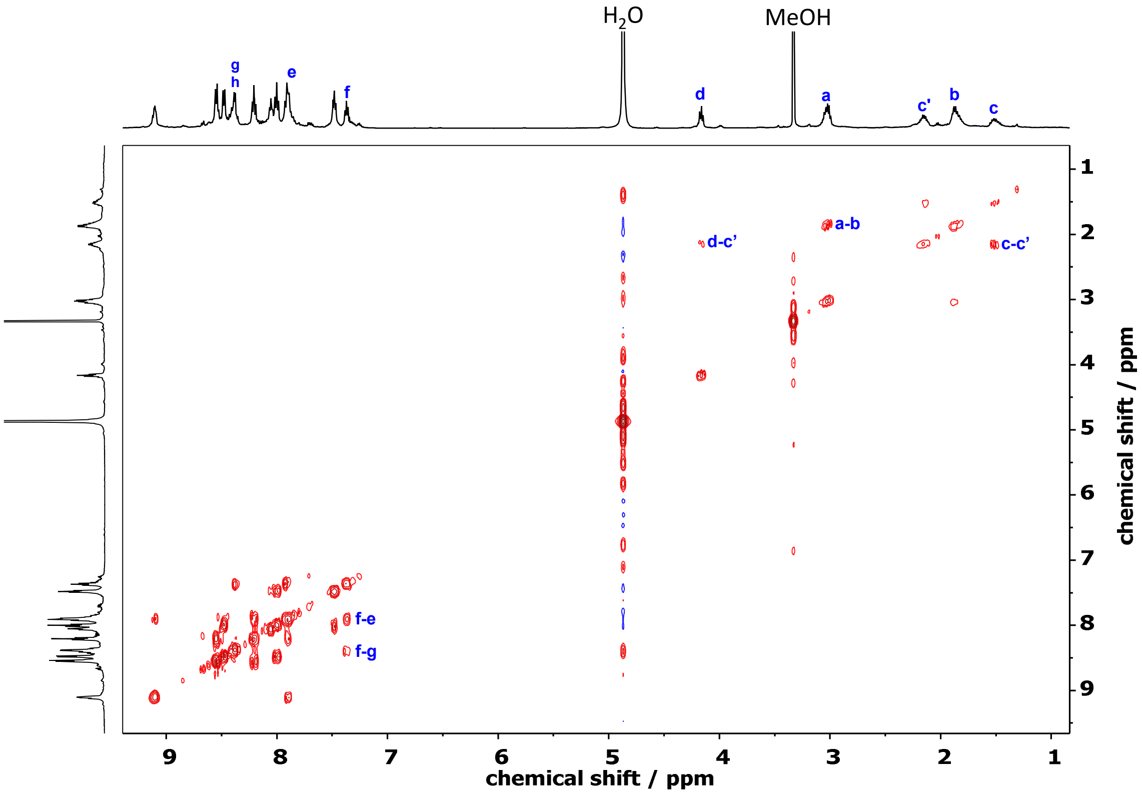

Figure S2. ^{1}H–^{1}H COSY spectrum (500 MHz) of **1** in CD_3OD at 298 K.

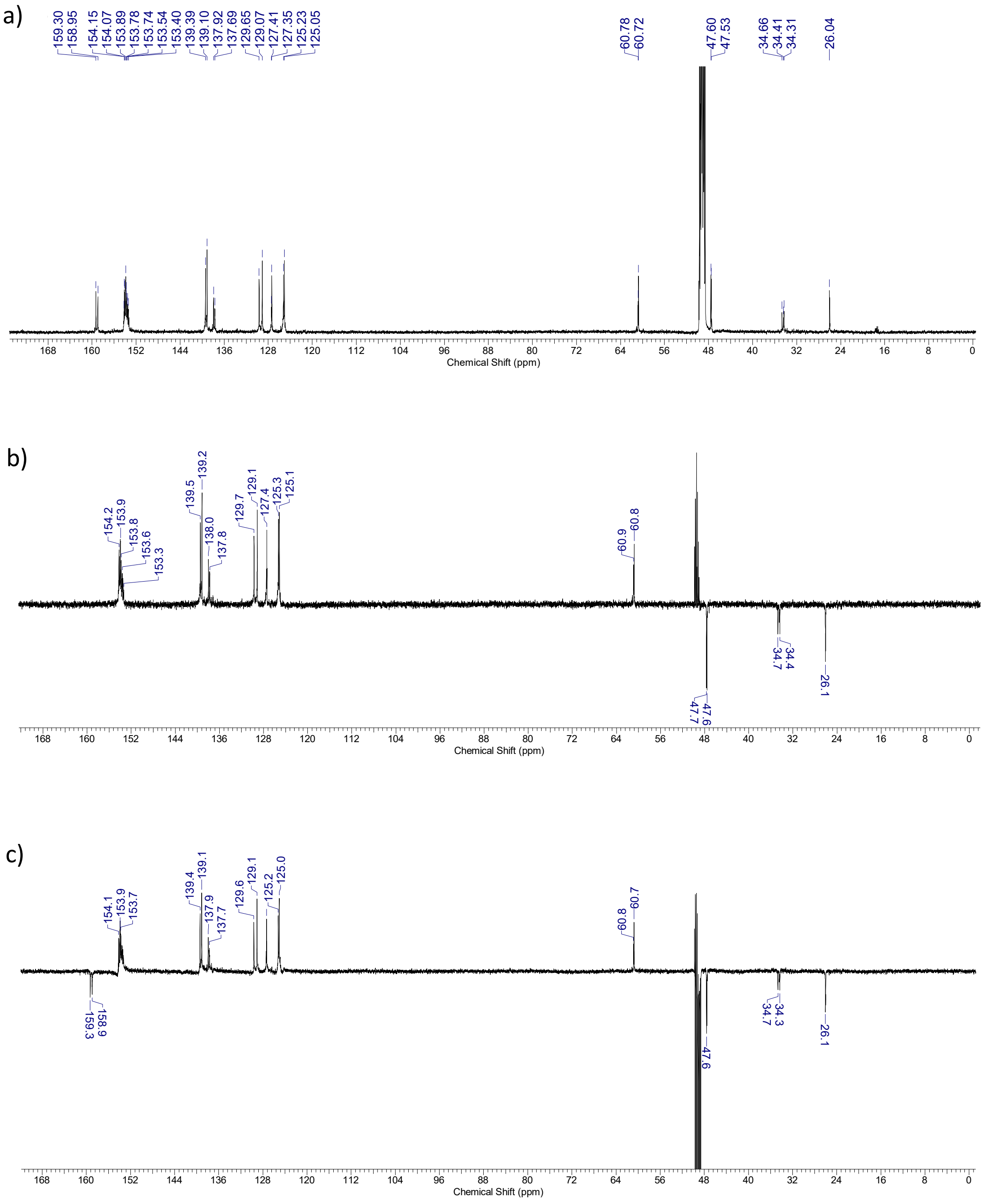

Figure S3. a) $^{13}C\{^1H\}$ NMR spectrum (126 MHz) of **1** in CD_3OD at 298 K. b) and c) $^{13}C\{^1H\}$ DEPT-135 and $^{13}C\{^1H\}$ DEPTQ-135 at 298 K, respectively.

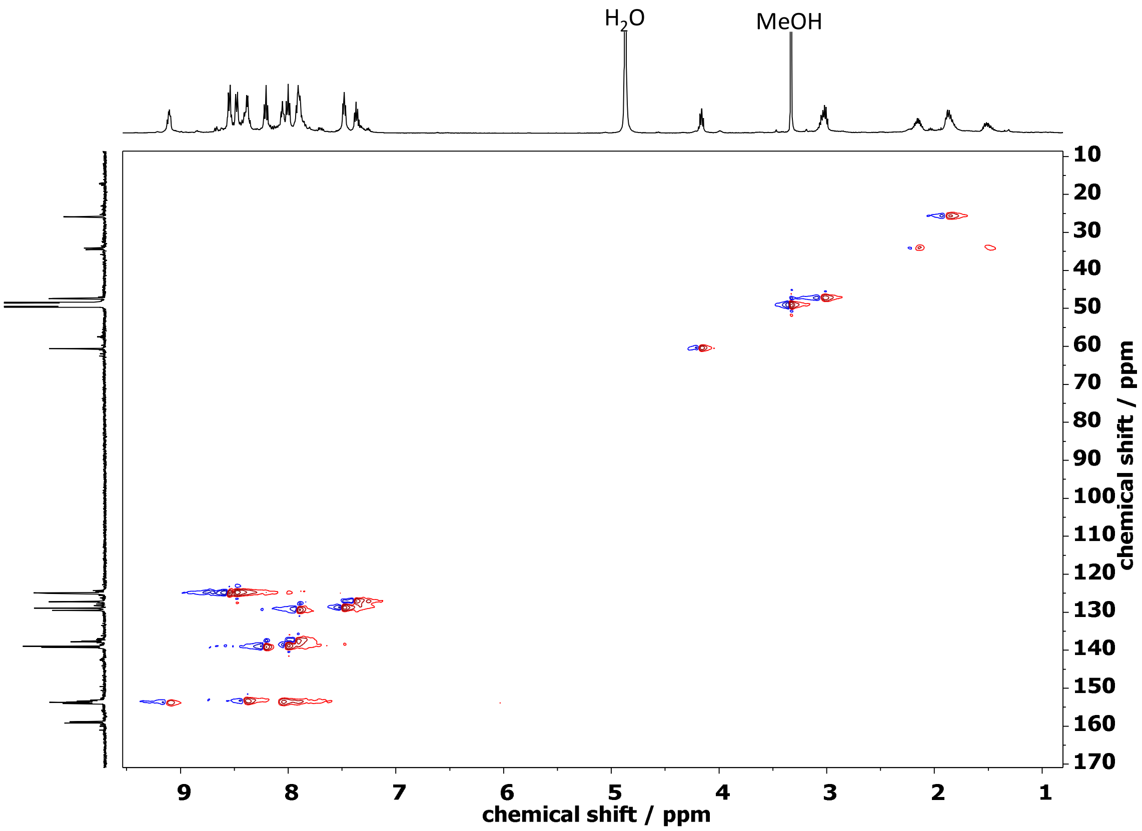

Figure S4. ^{1}H–^{13}C HSQC spectrum (500 MHz) of **1** in CD_3OD at 298 K.

2. Characterization of catalyst 1 by HRMS, UV/vis, and FTIR

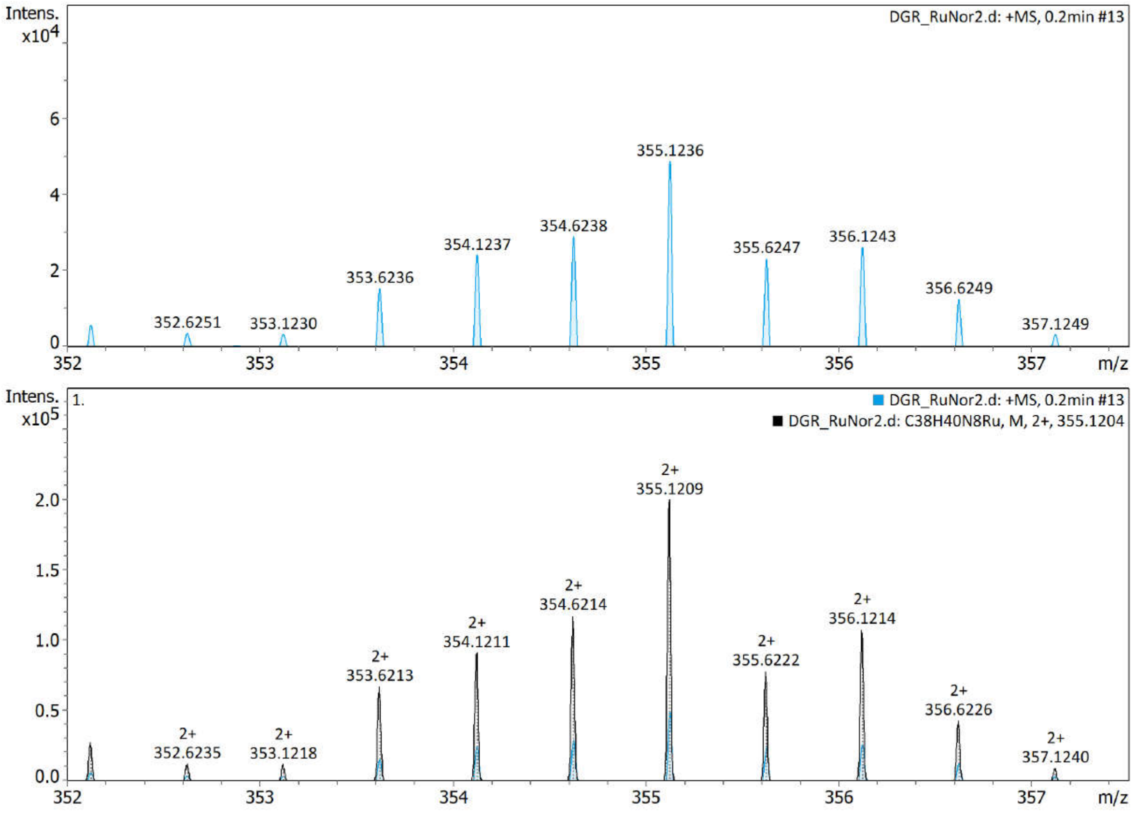

Figure S5. HRMS-ESI spectrum of **1** (M^{2+}; $[Ru(bpy)_2(Nor)_2]^{2+}$).

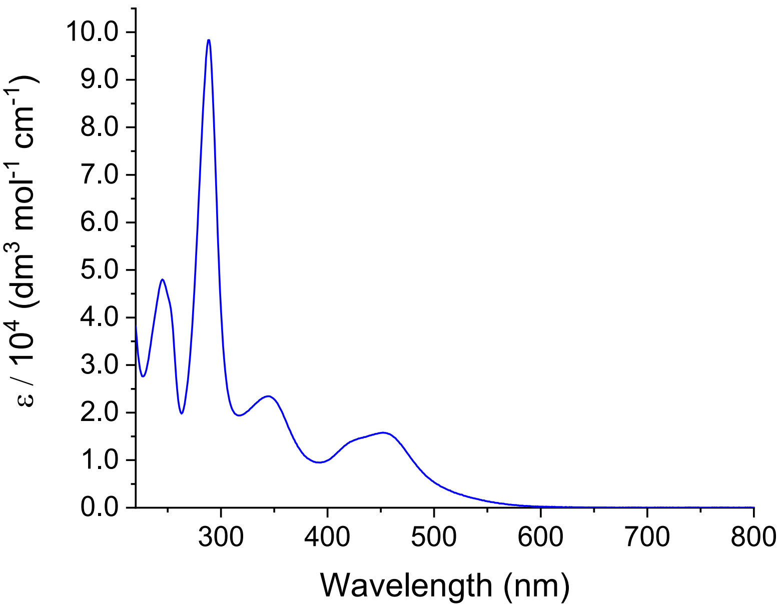

Figure S6. UV/vis absorption spectrum of **1** (20 µM) in phosphate-buffered D_2O (pD 8.5) at 298 K.

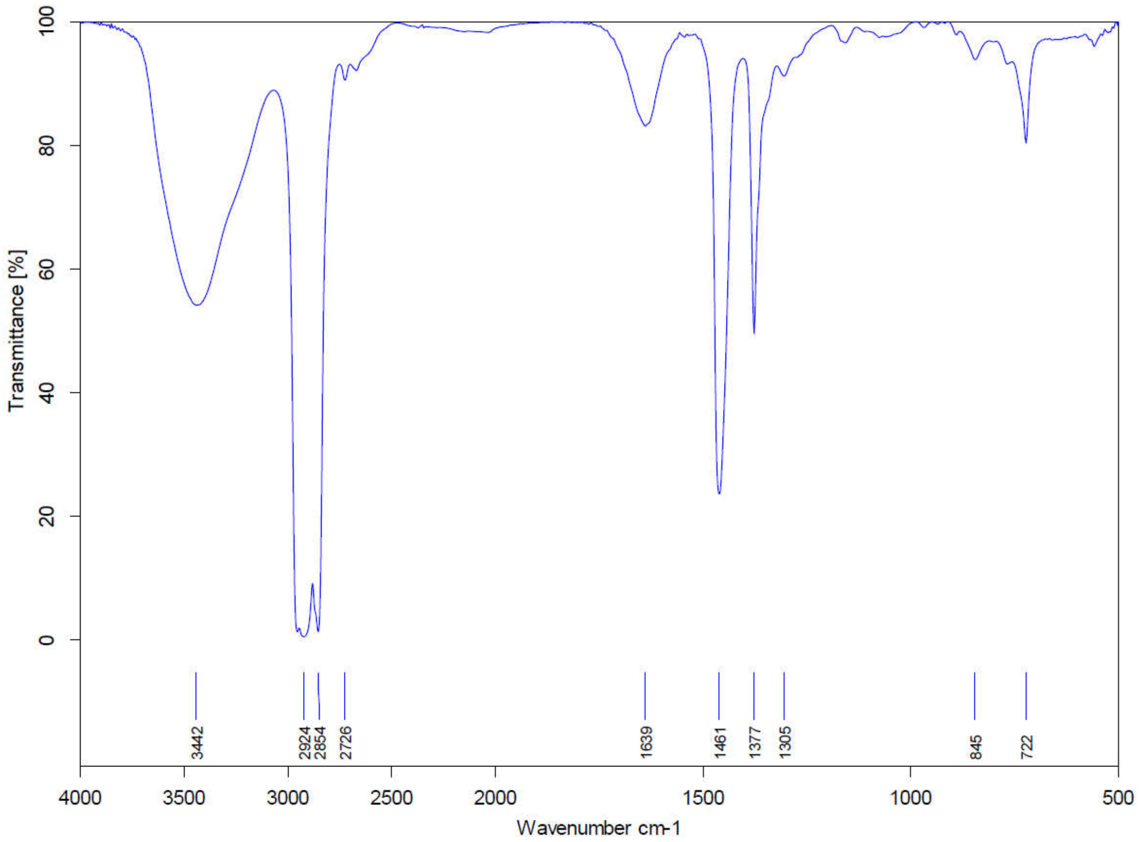

Figure S7. FTIR spectrum of **1**, dispersed in nujol.

3. Kinetic assays monitored by ^{1}H NMR spectroscopy

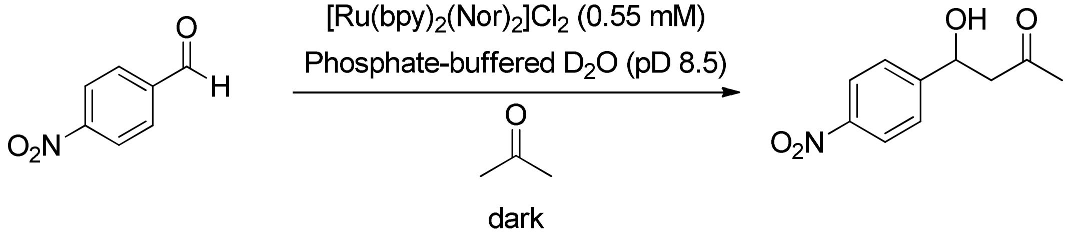

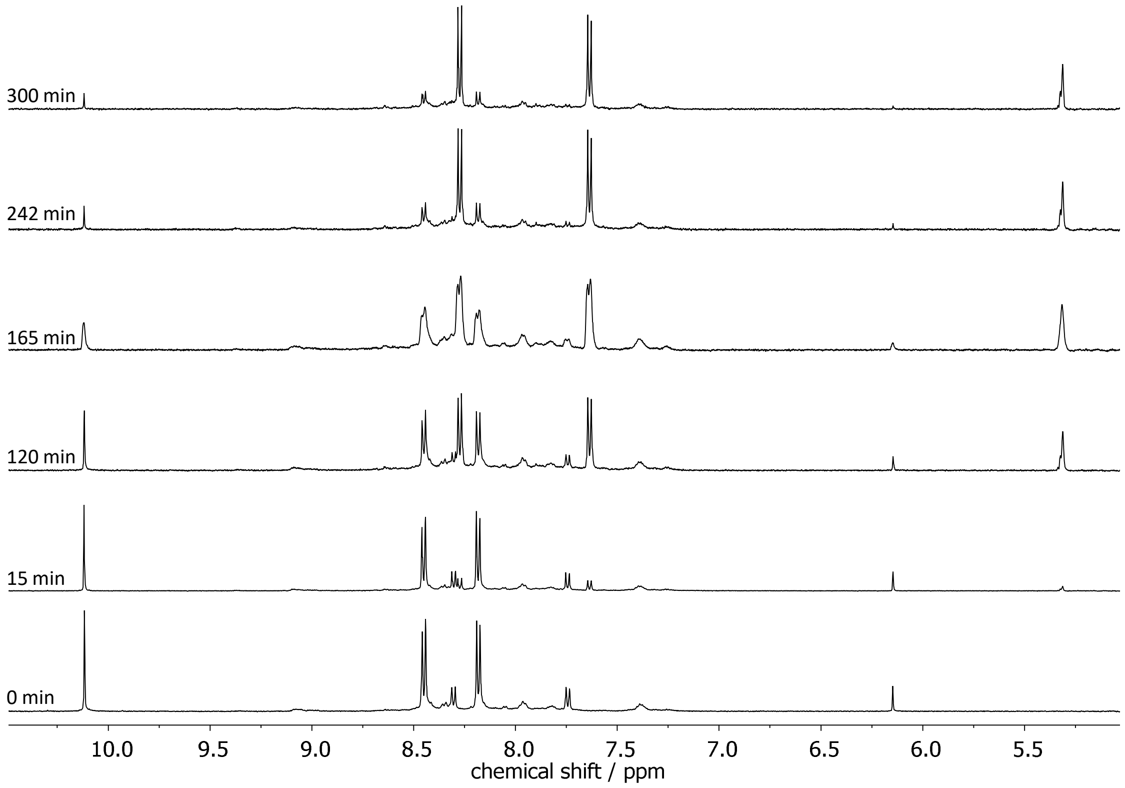

Aldol product formation:

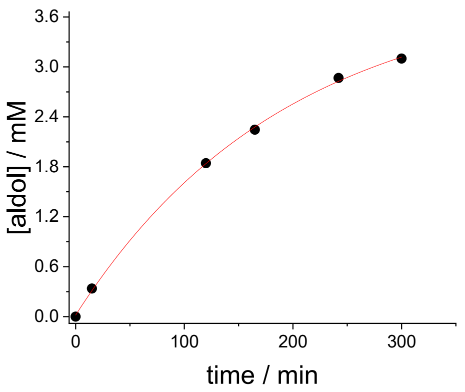

Pseudo-first-order rate constant: $\mathbf{k_{obs} = 6.5 \times 10^{-3}\ min^{-1}}$

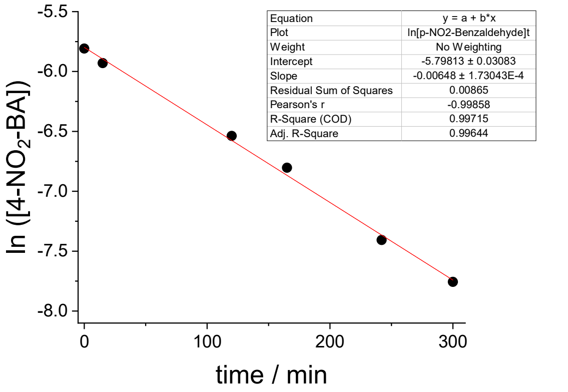

Figure S8. Partial ^{1}H NMR spectra for the aldol reaction between 4-nitrobenzaldehyde and acetone. Experimental conditions: **1** (0.55 mM), 4-nitrobenzaldehyde (3.6 mM), acetone (270 mM) in phosphate-buffered D_2O (45 mM at pD 8.5) at 298 K and different times.

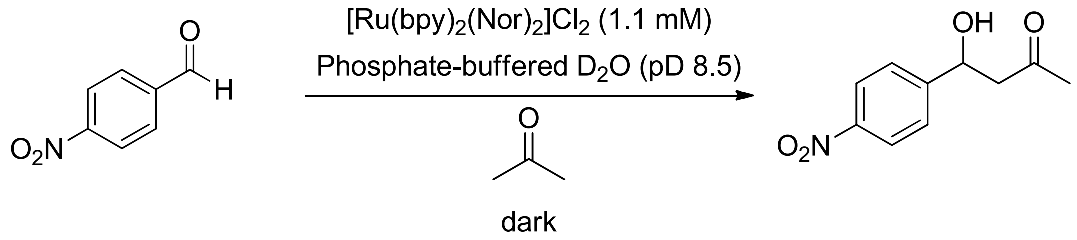

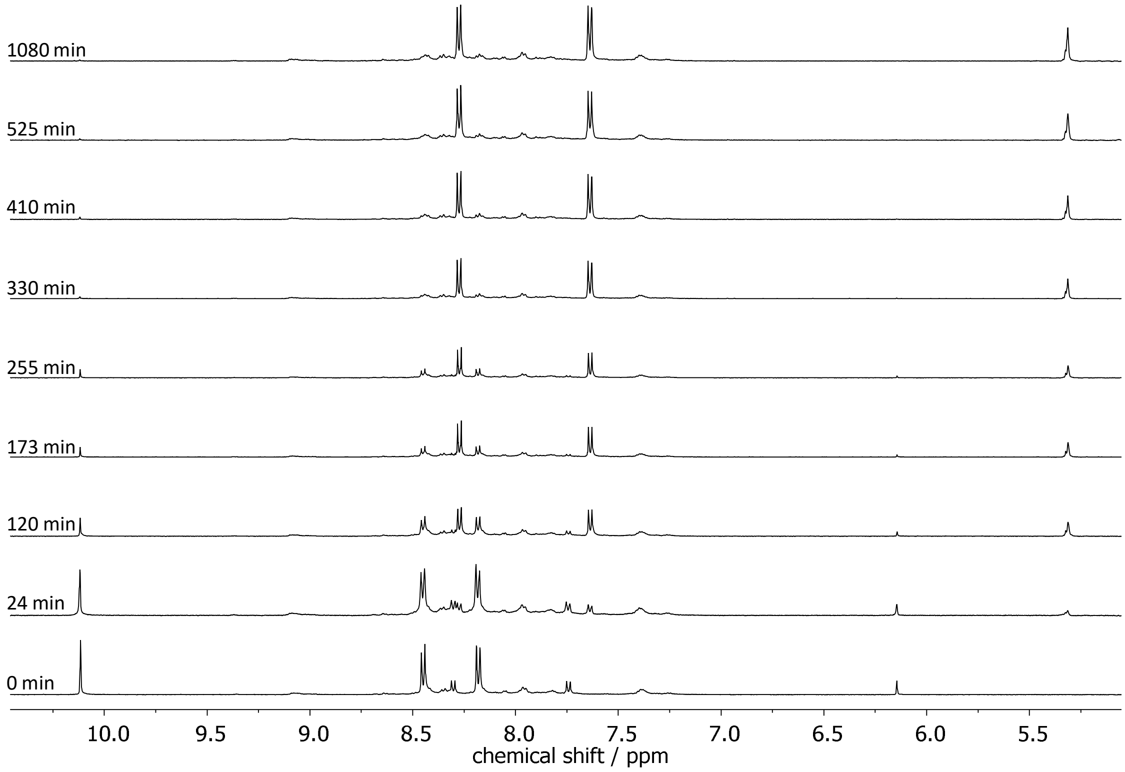

Aldol product formation:

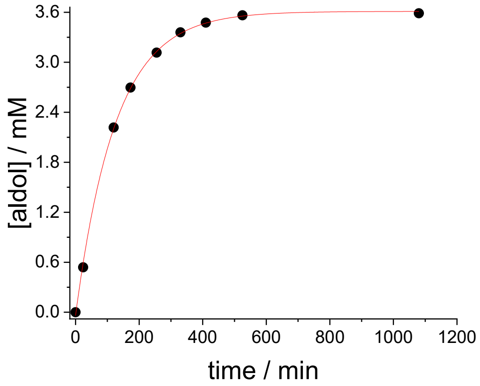

Pseudo-first-order rate constant: $\mathbf{k_{obs} = 8.5 \times 10^{-3}\ min^{-1}}$

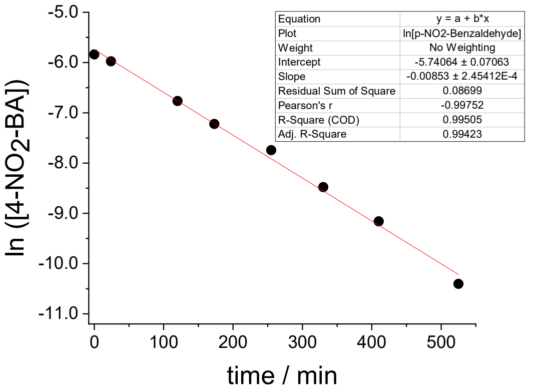

Figure S9. Partial ^{1}H NMR spectra for the aldol reaction between 4-nitrobenzaldehyde and acetone. Experimental conditions: **1** (1.1 mM), 4-nitrobenzaldehyde (3.6 mM), acetone (270 mM) in phosphate-buffered D_2O (45 mM at pD 8.5) at 298 K and different times.

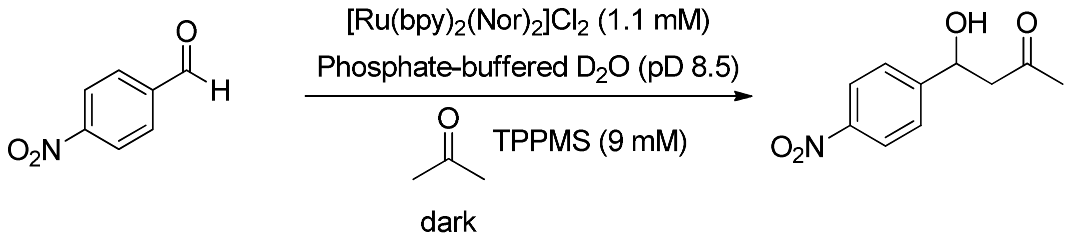

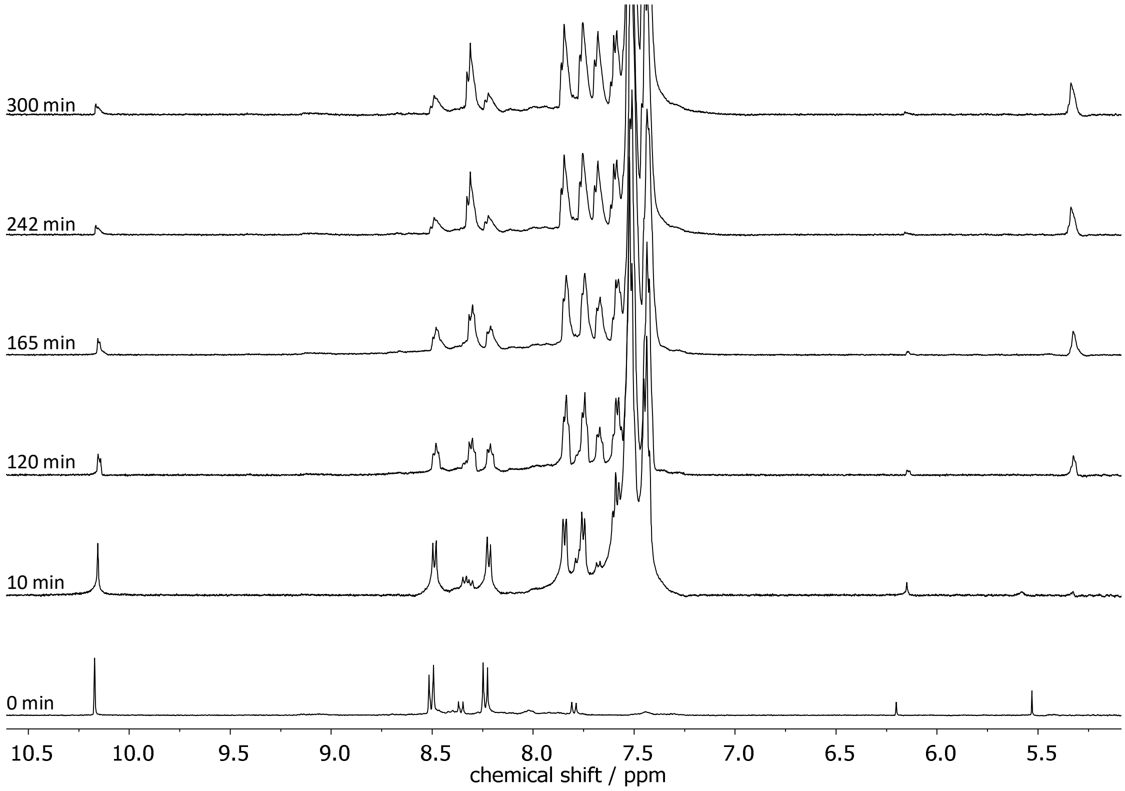

Aldol product formation:

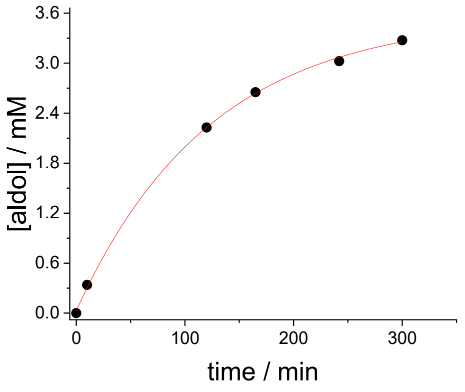

Pseudo-first-order rate constant: $\mathbf{k_{obs} = 7.8 \times 10^{-3}\ min^{-1}}$

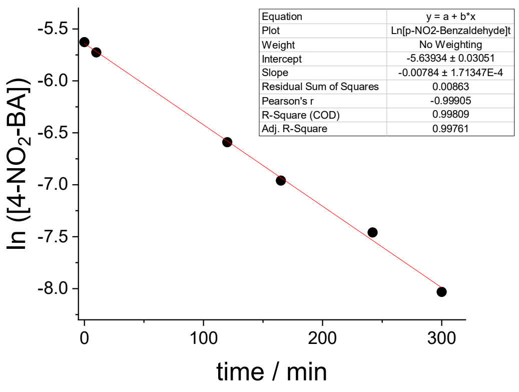

Figure S10. Partial ^{1}H NMR spectra for the aldol reaction between 4-nitrobenzaldehyde and acetone. Experimental conditions: **1** (1.1 mM), 4-nitrobenzaldehyde (3.6 mM), acetone (270 mM), 9 mM TPPMS in phosphate-buffered D_2O (45 mM at pD 8.5) at 298 K and different times.

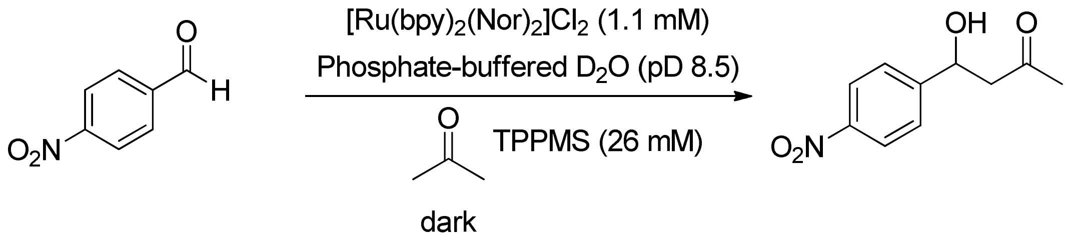

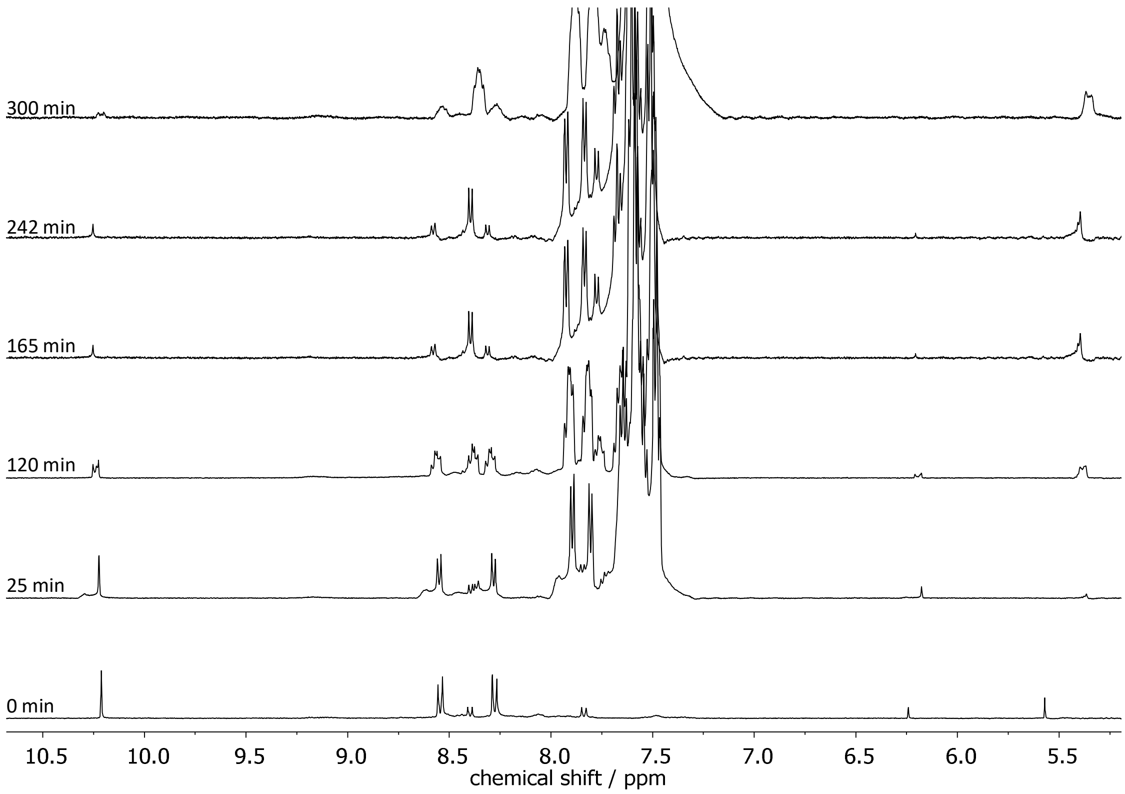

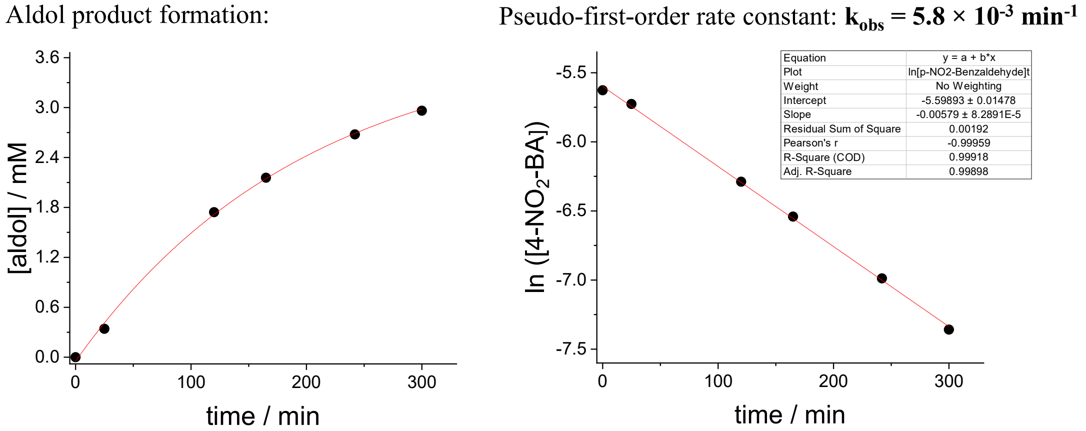

Figure S11. Partial ^1H NMR spectra for the aldol reaction between 4-nitrobenzaldehyde and acetone. Experimental conditions: **1** (1.1 mM), 4-nitrobenzaldehyde (3.6 mM), acetone (270 mM), 26 mM TPPMS in phosphate-buffered D_2O (45 mM at pD 8.5) at 298 K and different times.

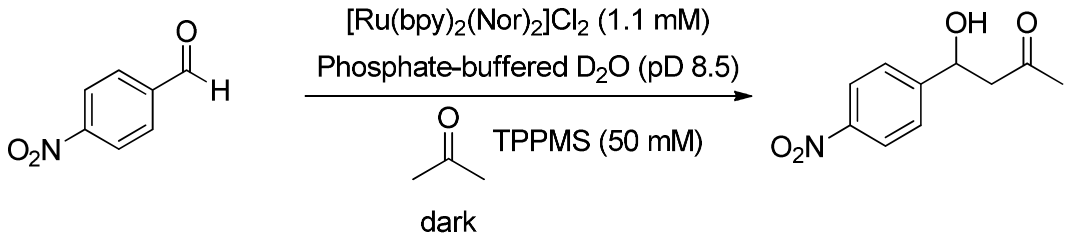

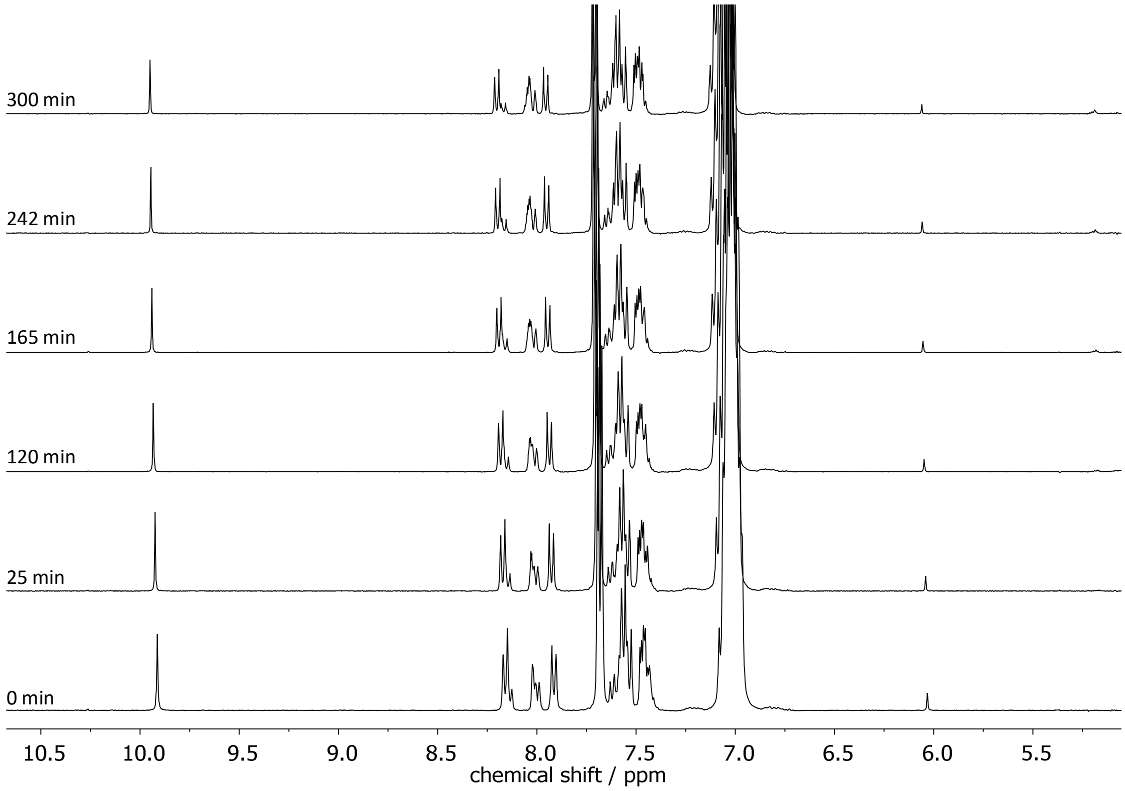

Aldol product formation:

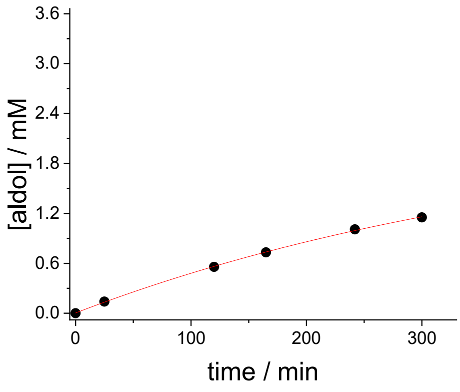

Pseudo-first-order rate constant: $\mathbf{k_{obs} = 1.3 \times 10^{-3}\ min^{-1}}$

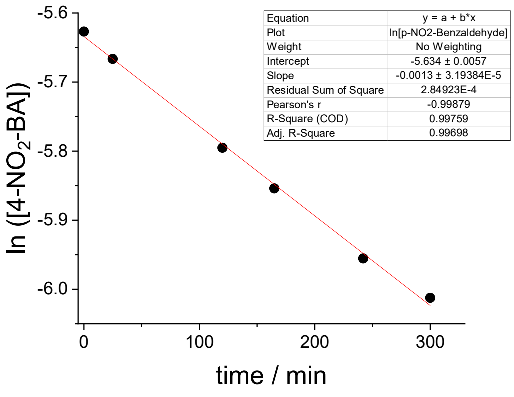

Figure S12. Partial ^{1}H NMR spectra for the aldol reaction between 4-nitrobenzaldehyde and acetone. Experimental conditions: **1** (1.1 mM), 4-nitrobenzaldehyde (3.6 mM), acetone (270 mM), 50 mM TPPMS in phosphate-buffered D_2O (45 mM at pD 8.5) at 298 K and different times.

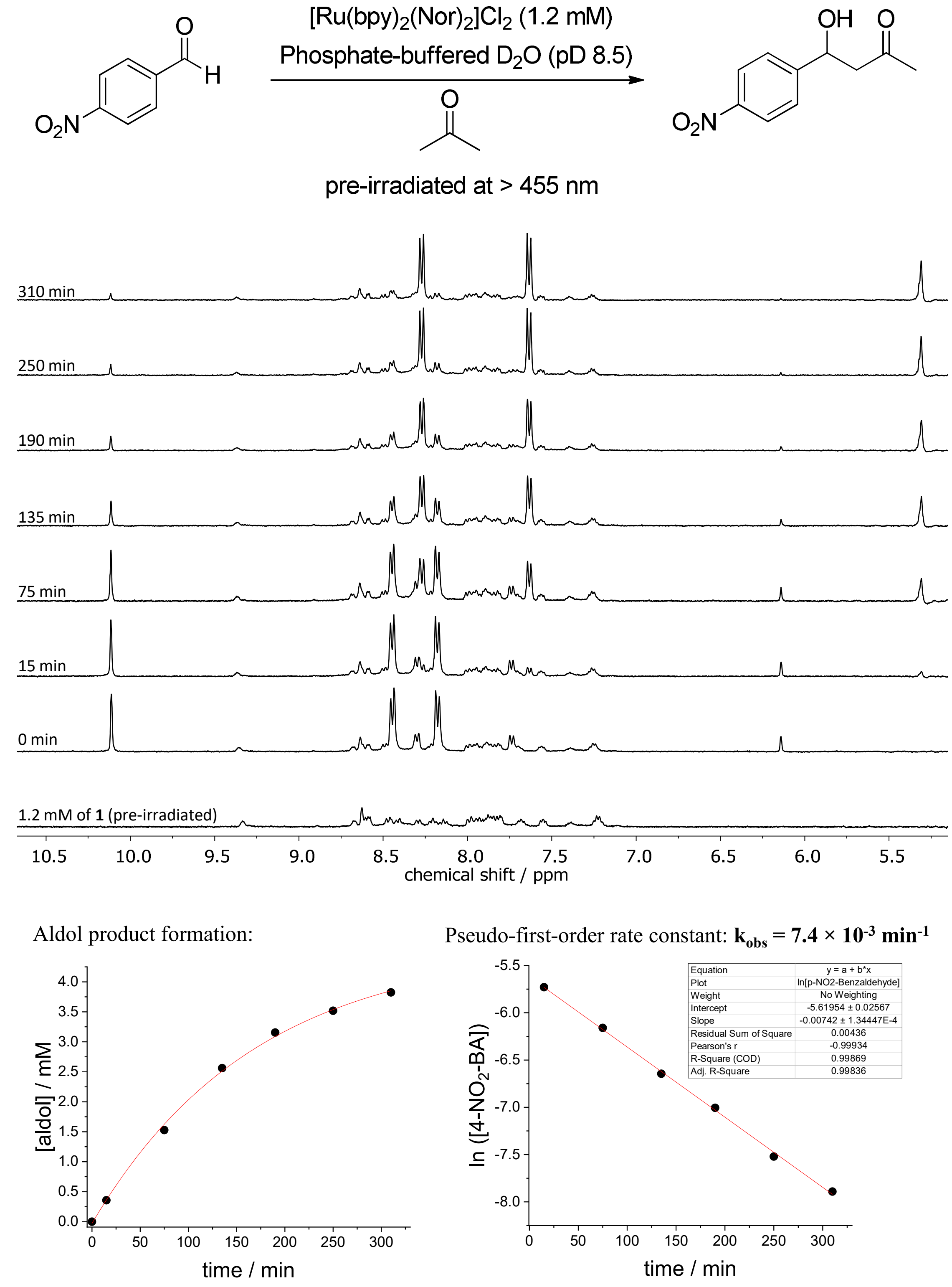

Figure S13. Partial ^{1}H NMR spectra for the aldol reaction between 4-nitrobenzaldehyde and acetone. Experimental conditions: **1** pre-irradiated 25 min at $\lambda > 455$ nm (1.2 mM), 4-nitrobenzaldehyde (4.0 mM), acetone (300 mM) in phosphate-buffered D_2O (50 mM at pD 8.5) at 298 K and different times.

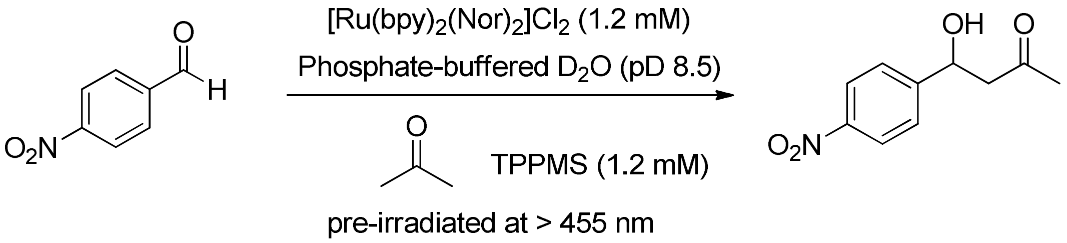

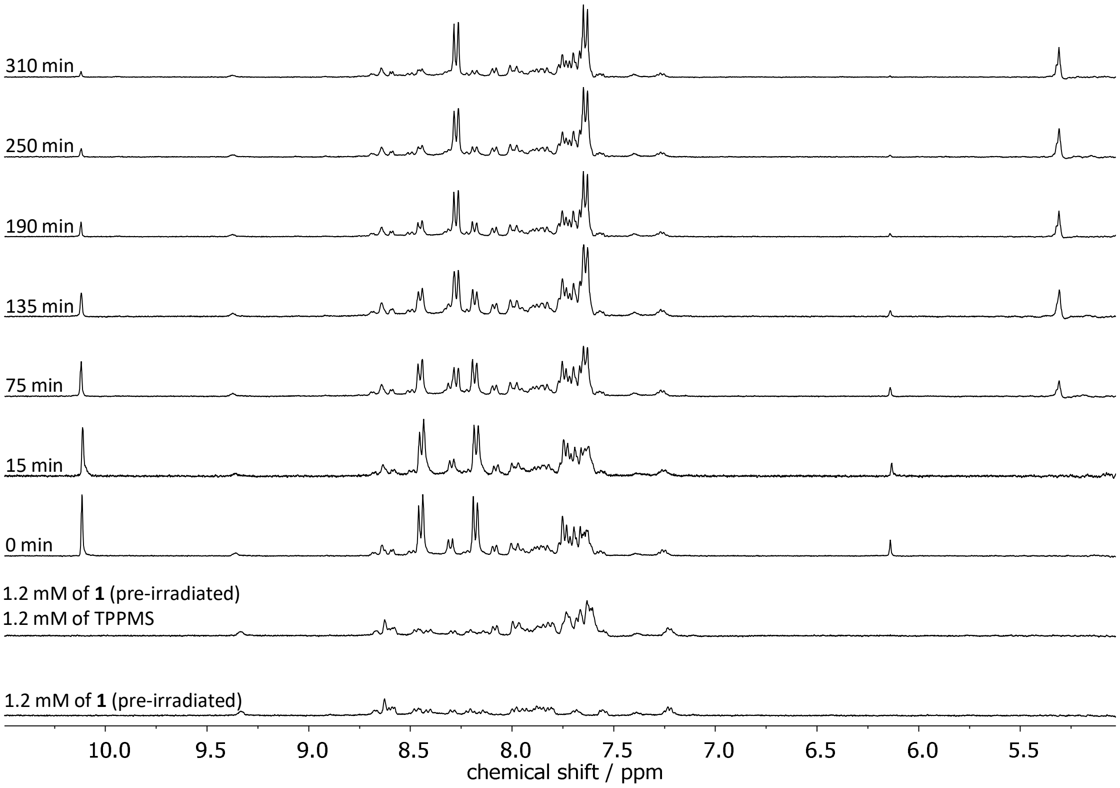

Aldol product formation:

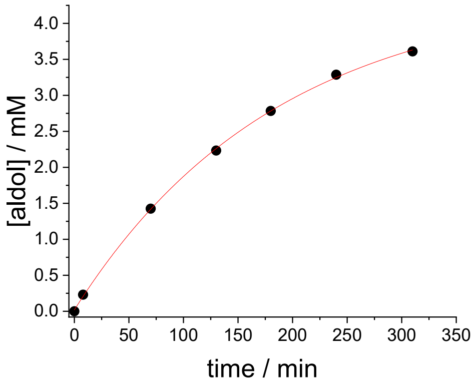

Pseudo-first-order rate constant: $\mathbf{k_{obs} = 7.0 \times 10^{-3}\ min^{-1}}$

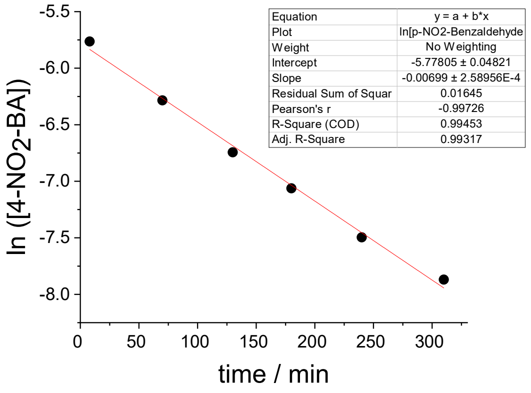

Figure S14. Partial ^{1}H NMR spectra for the aldol reaction between 4-nitrobenzaldehyde and acetone. Experimental conditions: **1** pre-irradiated 25 min at $\lambda > 455$ nm (1.2 mM), 4-nitrobenzaldehyde (4.0 mM), acetone (300 mM), 1.2 mM TPPMS in phosphate-buffered D_2O (50 mM at pD 8.5) at 298 K and different times.

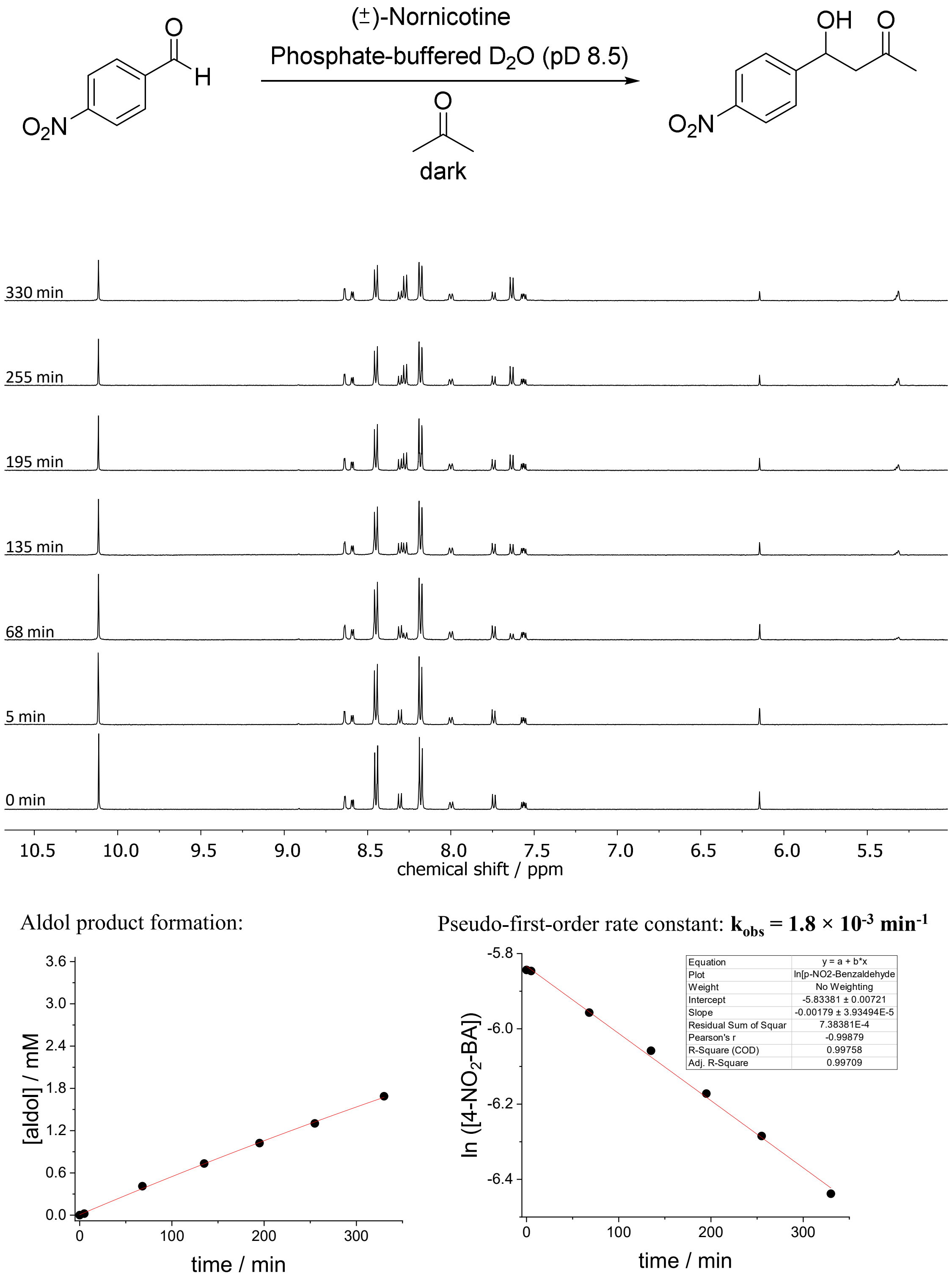

Figure S15. Partial ^{1}H NMR spectra for the aldol reaction between 4-nitrobenzaldehyde and acetone. Experimental conditions: (±)-nornicotine (1.1 mM), 4-nitrobenzaldehyde (3.6 mM), acetone (270 mM) in phosphate-buffered D_2O (45 mM at pD 8.5) at 298 K and different times.

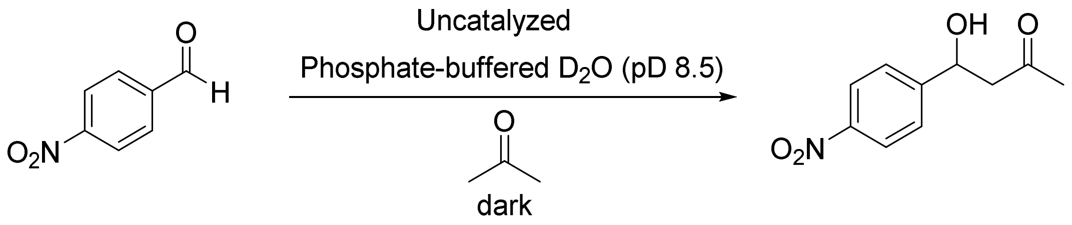

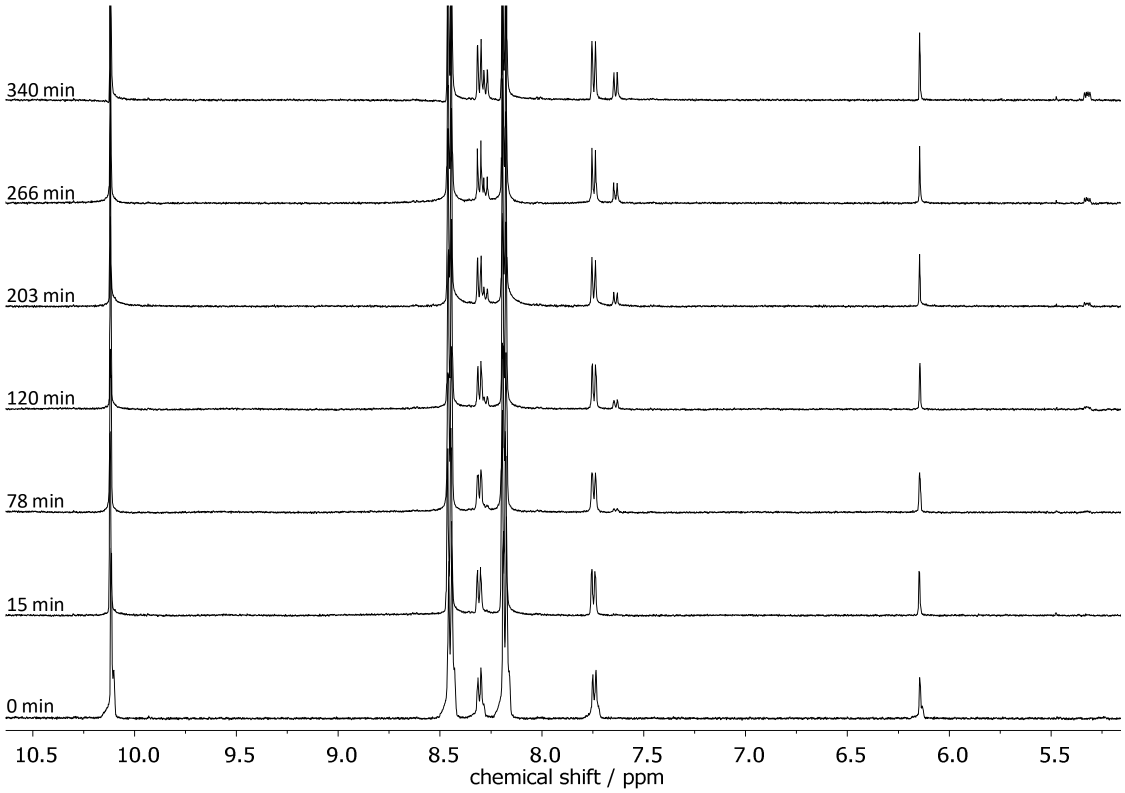

Aldol product formation:

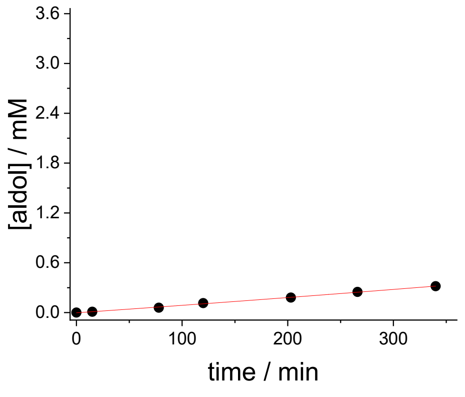

Pseudo-first-order rate constant: $\mathbf{k_{obs} = 3.4 \times 10^{-4}\ min^{-1}}$

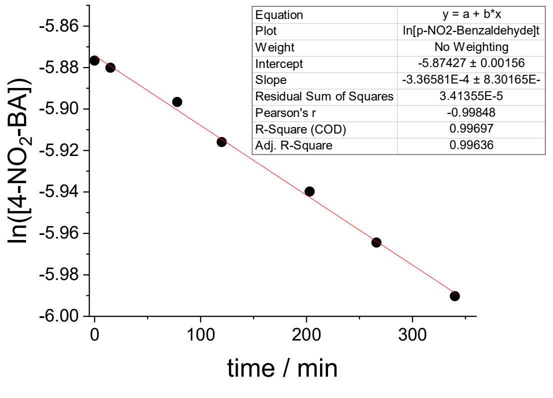

Figure S16. Partial ^{1}H NMR spectra for the aldol reaction between 4-nitrobenzaldehyde and acetone. Experimental conditions: 4-nitrobenzaldehyde (3.6 mM), acetone (270 mM) in phosphate-buffered D_2O (45 mM at pD 8.5) at 298 K and different times.

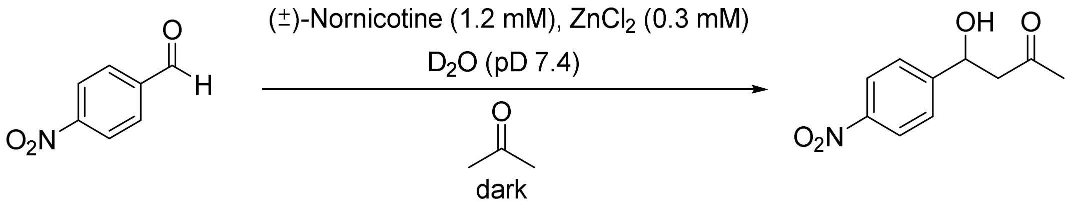

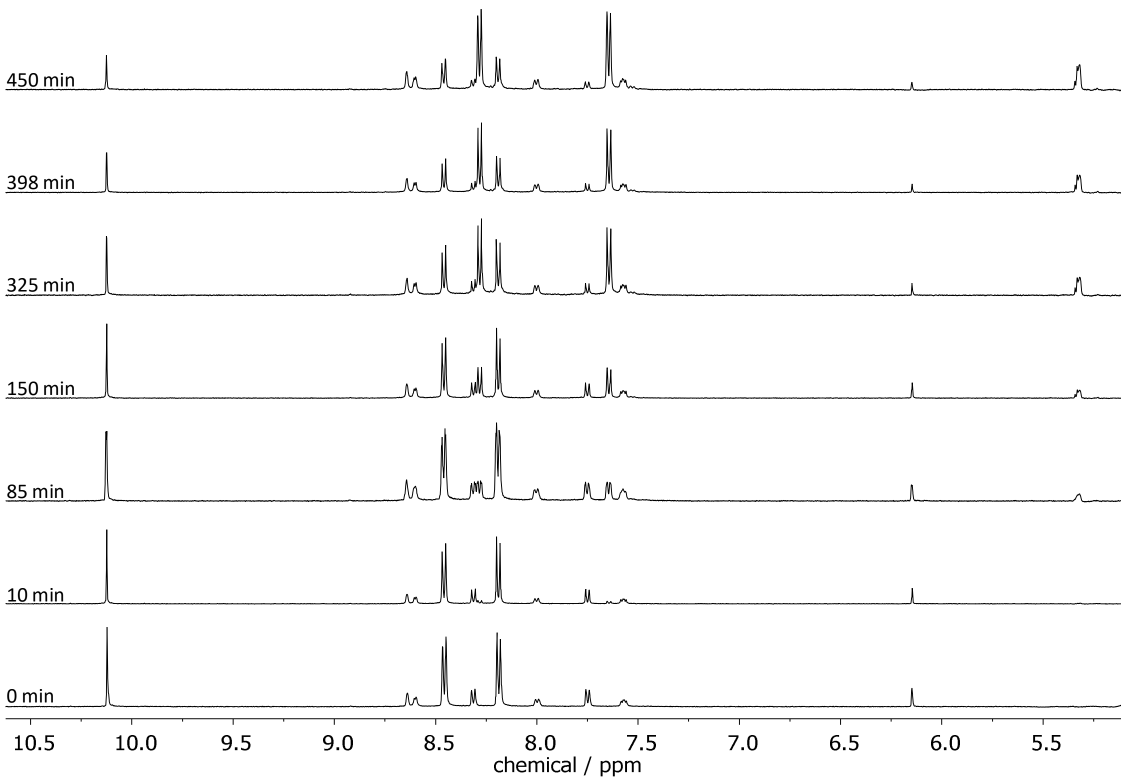

Aldol product formation:

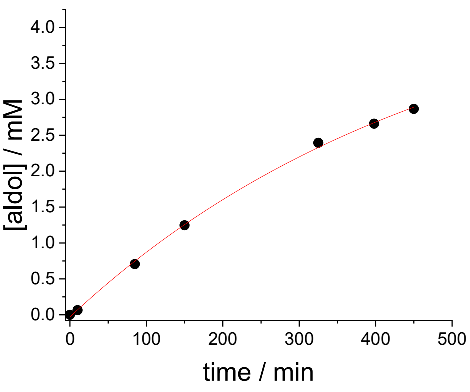

Pseudo-first-order rate constant: $\mathbf{k_{obs} = 2.8 \times 10^{-3}\ min^{-1}}$

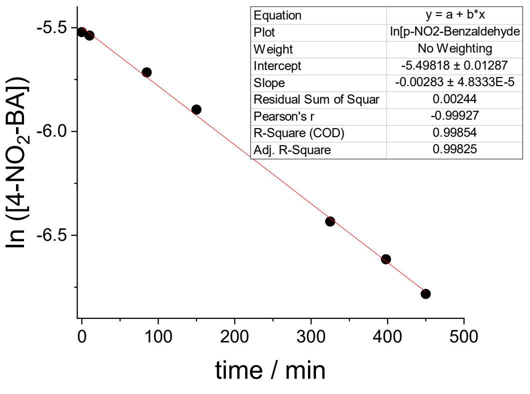

Figure S17. Partial ^{1}H NMR spectra for the aldol reaction between 4-nitrobenzaldehyde and acetone. Experimental conditions: (±)-nornicotine (1.2 mM), $ZnCl_2$ (0.3 mM), 4-nitrobenzaldehyde (4.0 mM), acetone (300 mM) in D_2O (pD 7.4) at 298 K and different times.

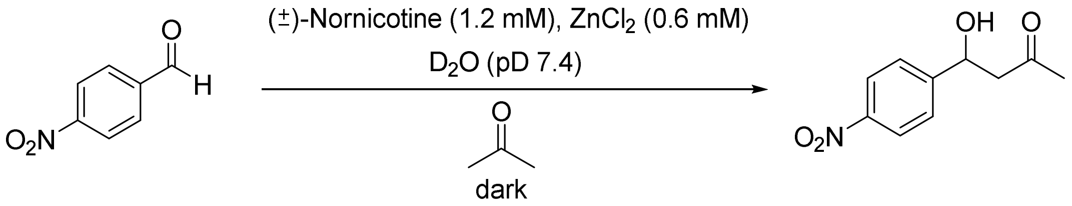

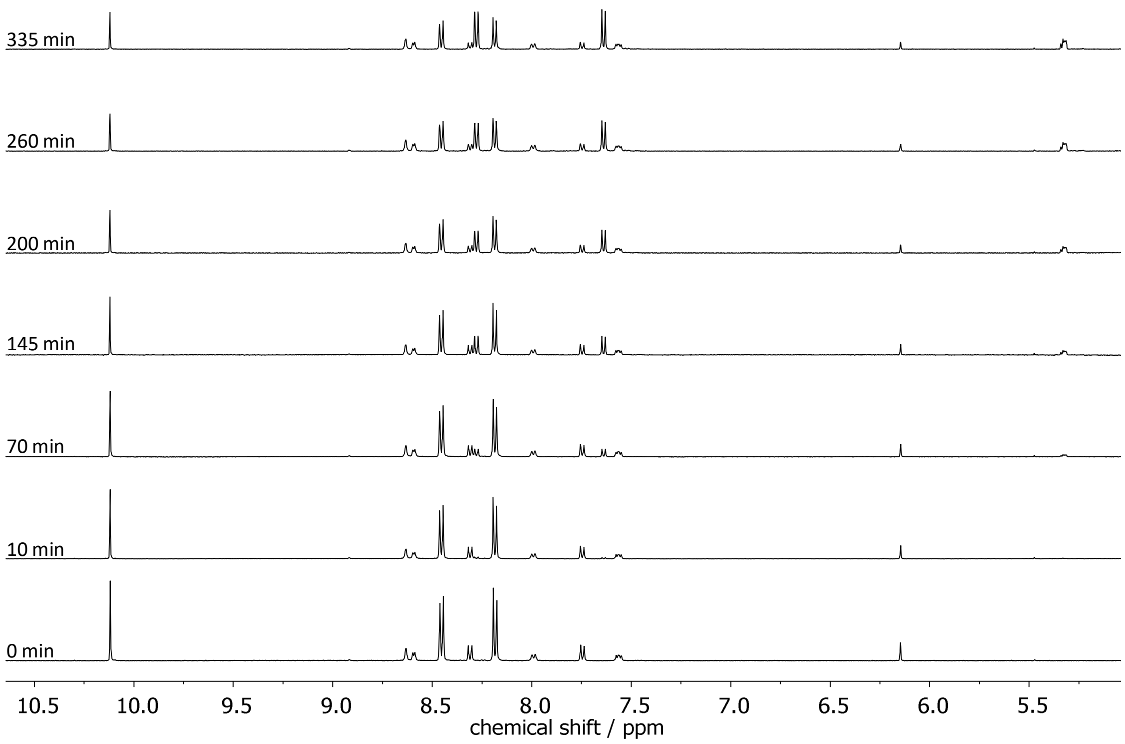

Aldol product formation:

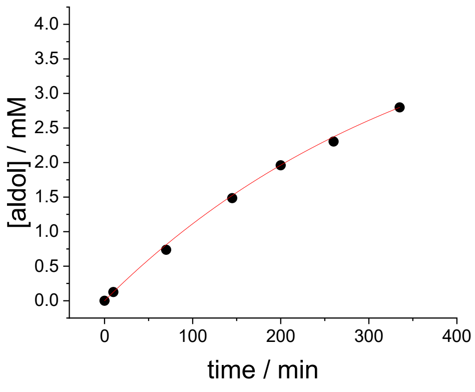

Pseudo-first-order rate constant: $\mathbf{k_{obs} = 3.4 \times 10^{-3}\ min^{-1}}$

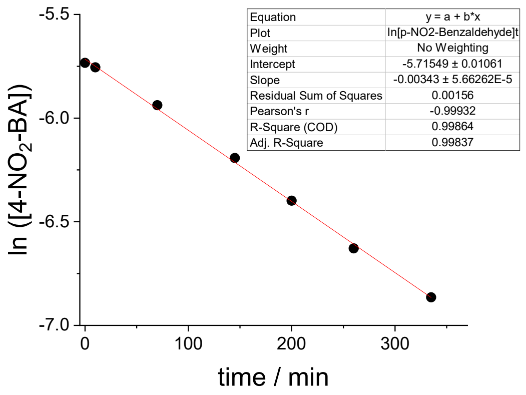

Figure S18. Partial ^{1}H NMR spectra for the aldol reaction between 4-nitrobenzaldehyde and acetone. Experimental conditions: (±)-nornicotine (1.2 mM), $ZnCl_2$ (0.6 mM), 4-nitrobenzaldehyde (4.0 mM), acetone (300 mM) in D_2O (pD 7.4) at 298 K and different times.

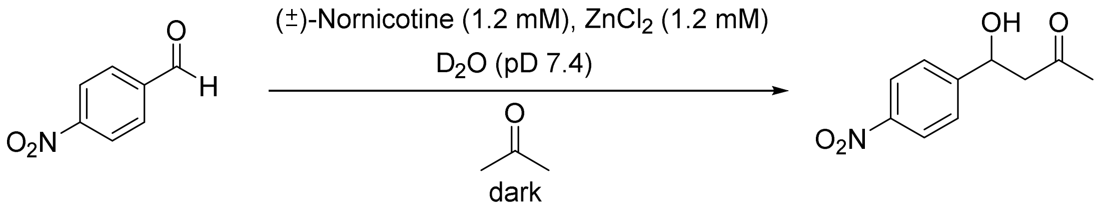

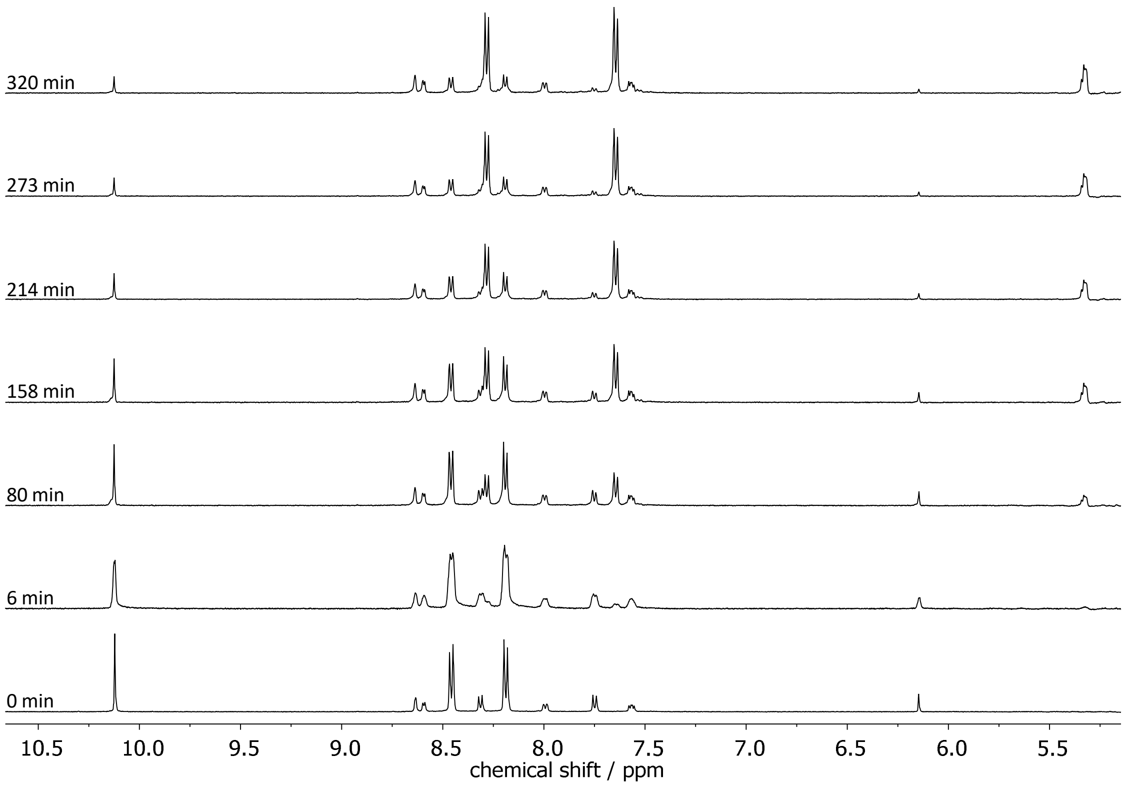

Aldol product formation:

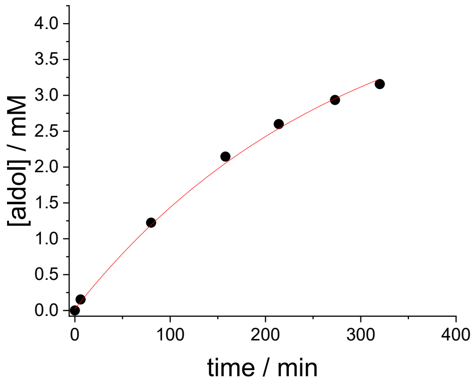

Pseudo-first-order rate constant: $\mathbf{k_{obs} = 4.9 \times 10^{-3}\ min^{-1}}$

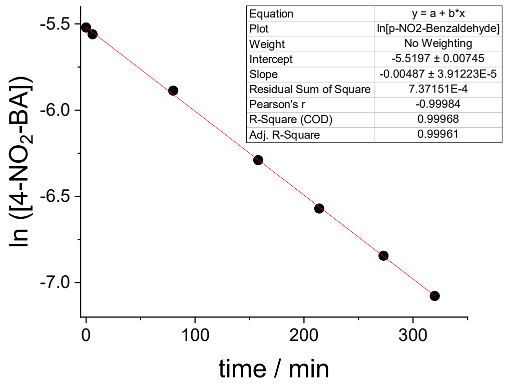

Figure S19. Partial ^{1}H NMR spectra for the aldol reaction between 4-nitrobenzaldehyde and acetone. Experimental conditions: (±)-nornicotine (1.2 mM), $ZnCl_2$ (1.2 mM), 4-nitrobenzaldehyde (4.0 mM), acetone (300 mM) in D_2O (pD 7.4) at 298 K and different times.

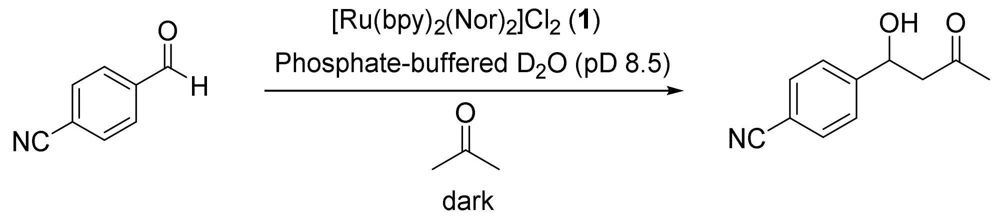

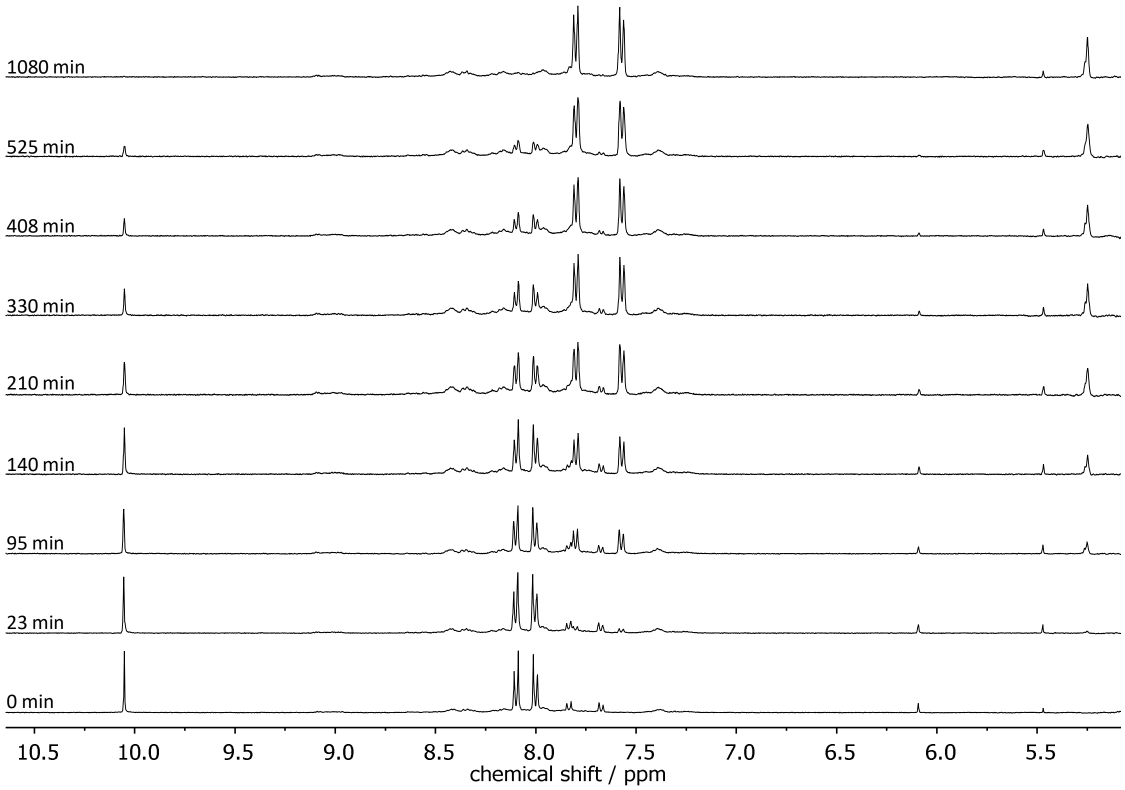

Aldol product formation:

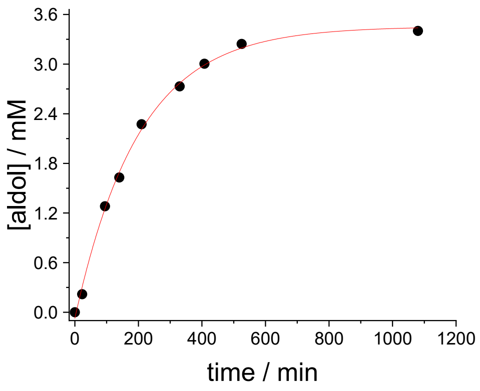

Pseudo-first-order rate constant: $\mathbf{k_{obs} = 4.4 \times 10^{-3}\ min^{-1}}$

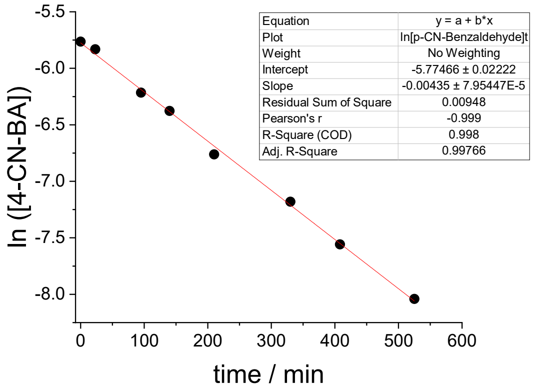

Figure S20. Partial ^{1}H NMR spectra for the aldol reaction between 4-cyanobenzaldehyde and acetone. Experimental conditions: **1** (1.1 mM), 4-cyanobenzaldehyde (3.6 mM), acetone (270 mM) in phosphate-buffered D_2O (45 mM at pD 8.5) at 298 K and different times.

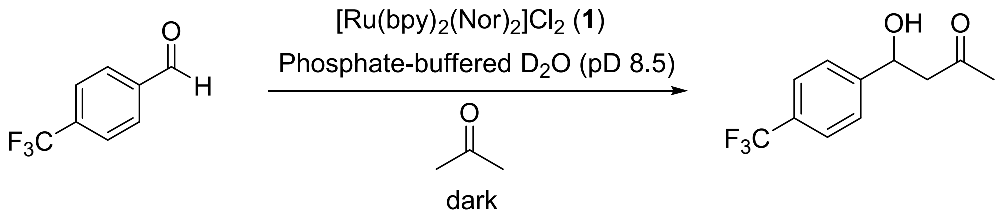

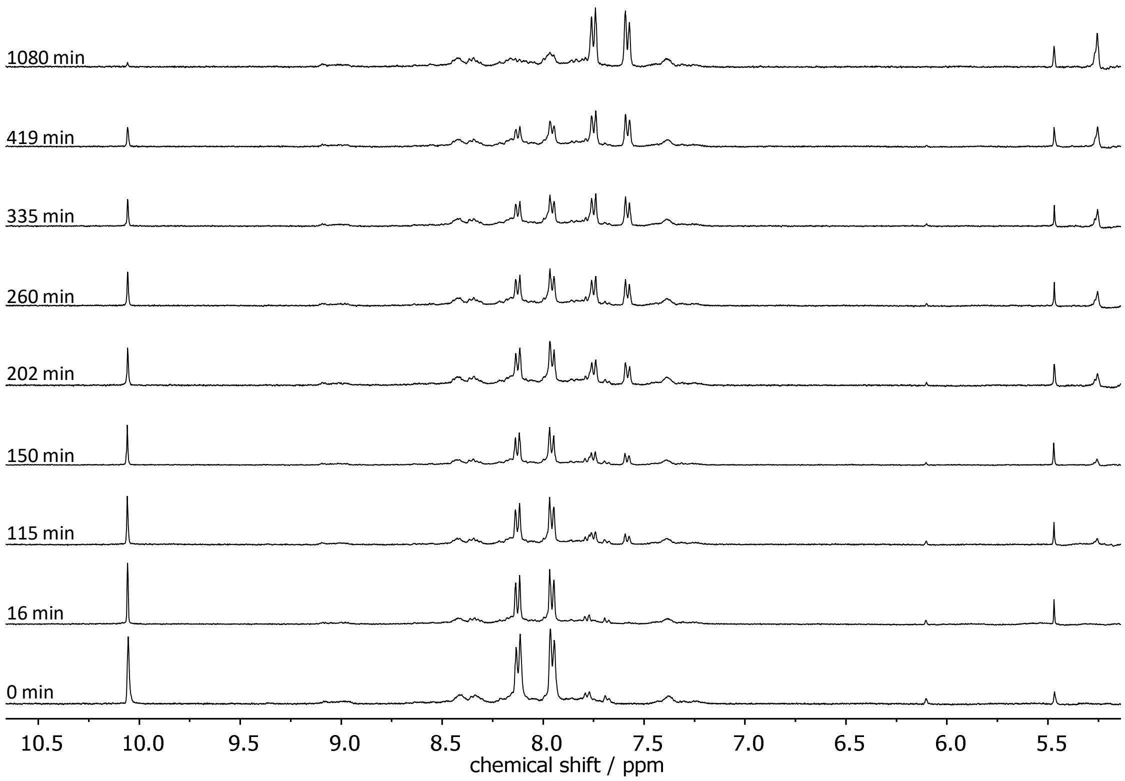

Aldol product formation:

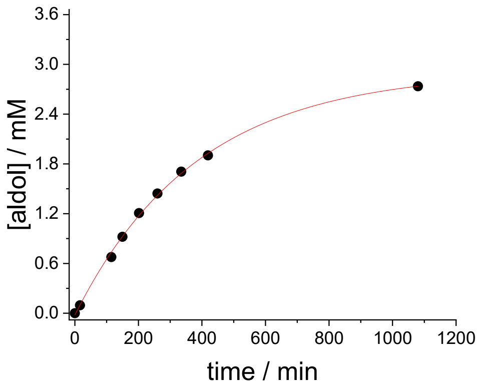

Pseudo-first-order rate constant: $\mathbf{k_{obs} = 1.9 \times 10^{-3}\ min^{-1}}$

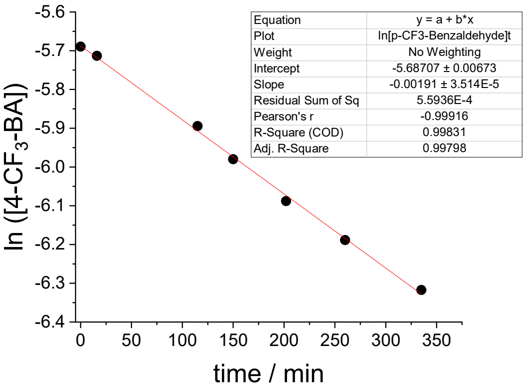

Figure S21. Partial ^{1}H NMR spectra for the aldol reaction between 4-(trifluoromethyl)benzaldehyde and acetone. Experimental conditions: **1** (1.1 mM), 4-(trifluoromethyl)benzaldehyde (3.6 mM), acetone (270 mM) in phosphate-buffered D_2O (45 mM at pD 8.5) at 298 K and different times.

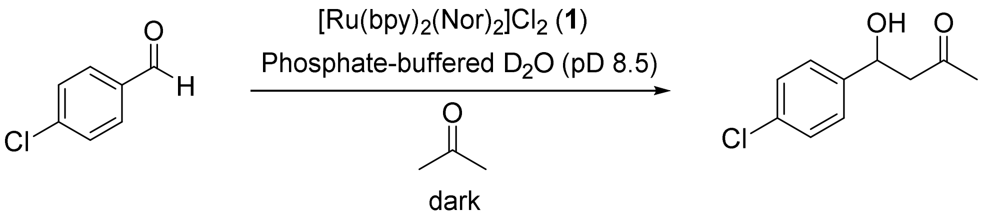

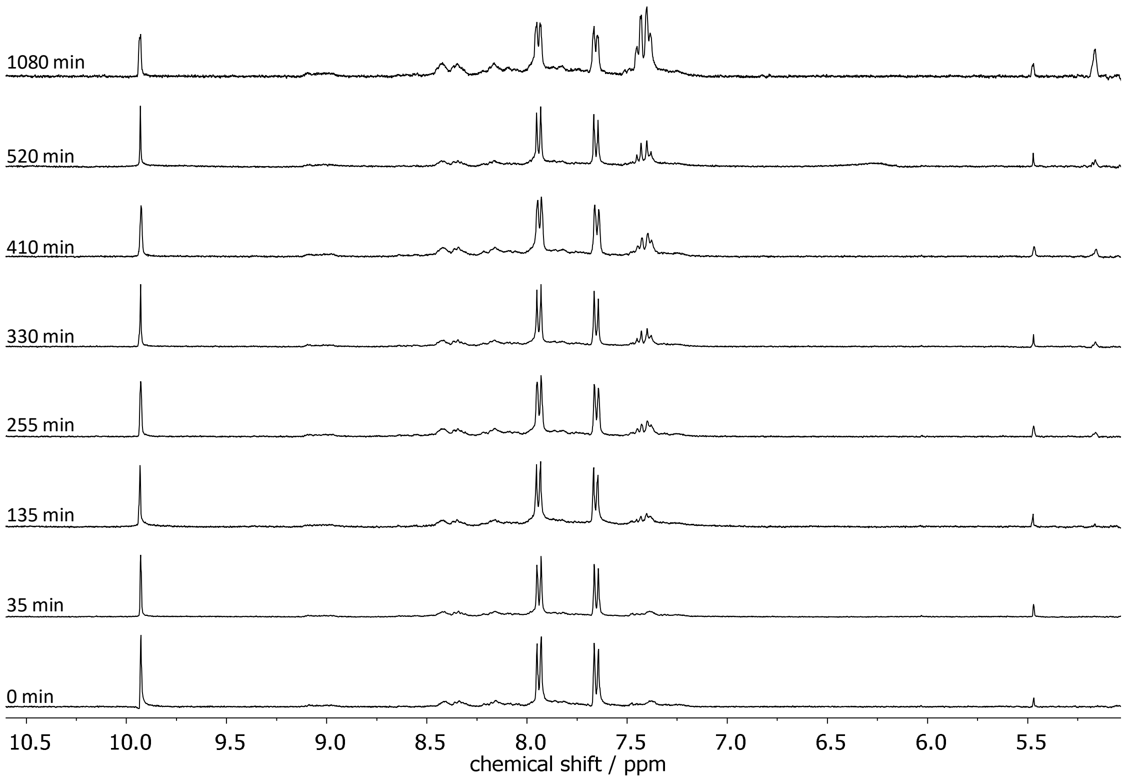

Aldol product formation:

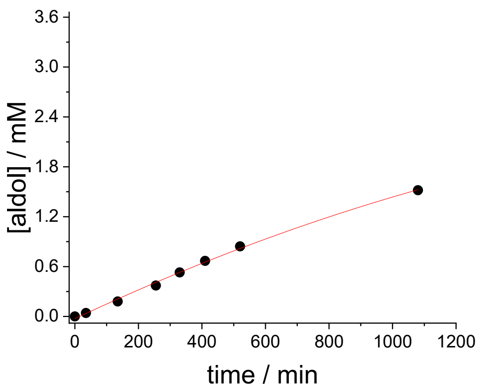

Pseudo-first-order rate constant: $\mathbf{k_{obs} = 5.1 \times 10^{-4}\ min^{-1}}$

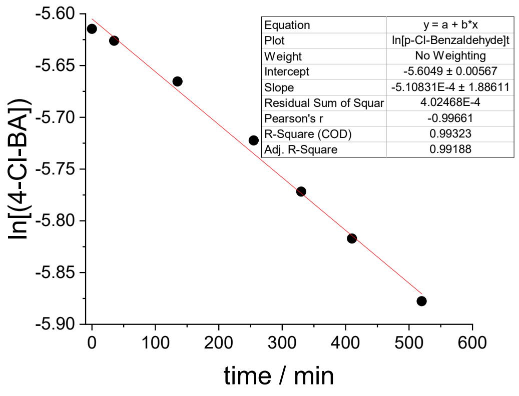

Figure S22. Partial ^{1}H NMR spectra for the aldol reaction between 4-chlorobenzaldehyde and acetone. Experimental conditions: **1** (1.1 mM), 4-chlorobenzaldehyde (3.6 mM), acetone (270 mM) in phosphate-buffered D_2O (45 mM at pD 8.5) at 298 K and different times.

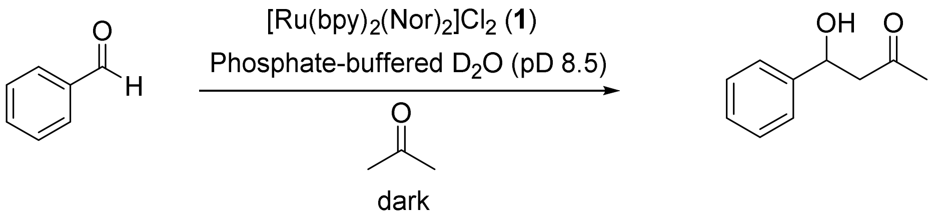

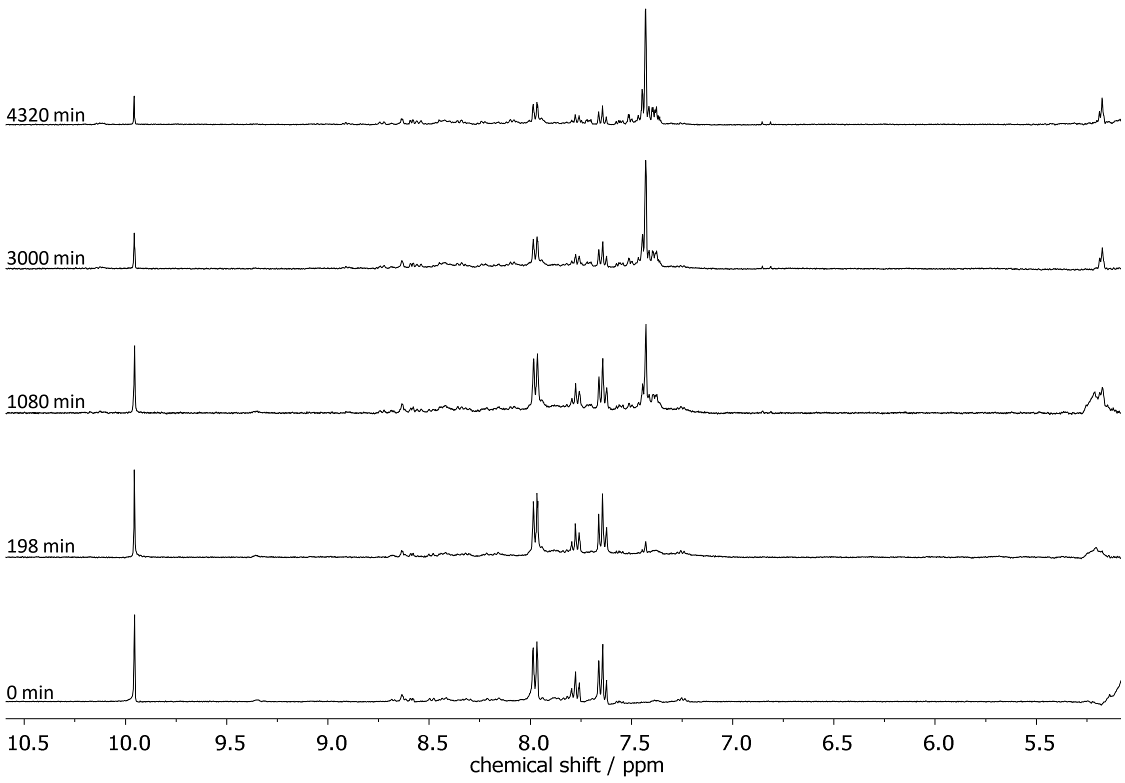

Aldol product formation:

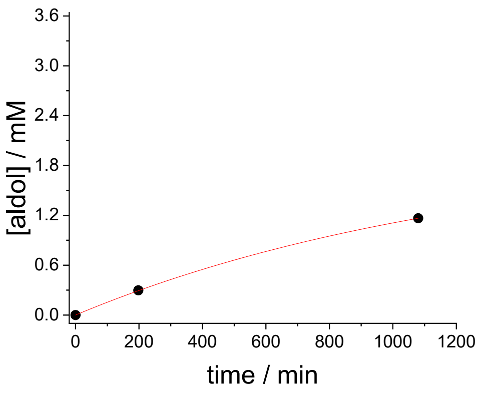

Pseudo-first-order rate constant: $\mathbf{k_{obs} = 3.4 \times 10^{-4}\ min^{-1}}$

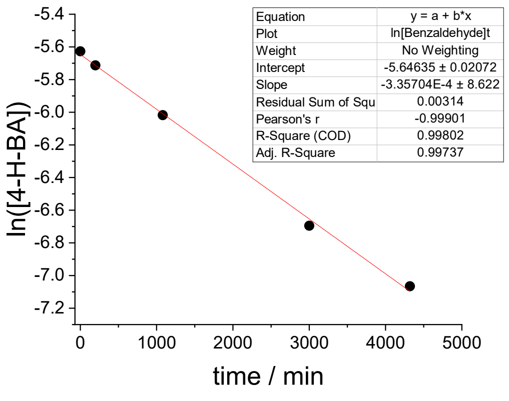

Figure S23. Partial ^{1}H NMR spectra for the aldol reaction between benzaldehyde and acetone. Experimental conditions: **1** (1.1 mM), benzaldehyde (3.6 mM), acetone (270 mM) in phosphate-buffered D_2O (45 mM at pD 8.5) at 298 K and different times.

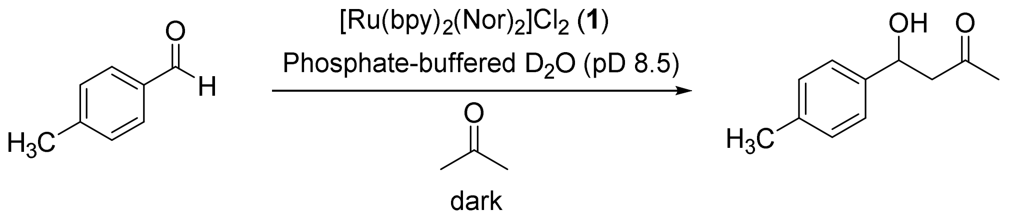

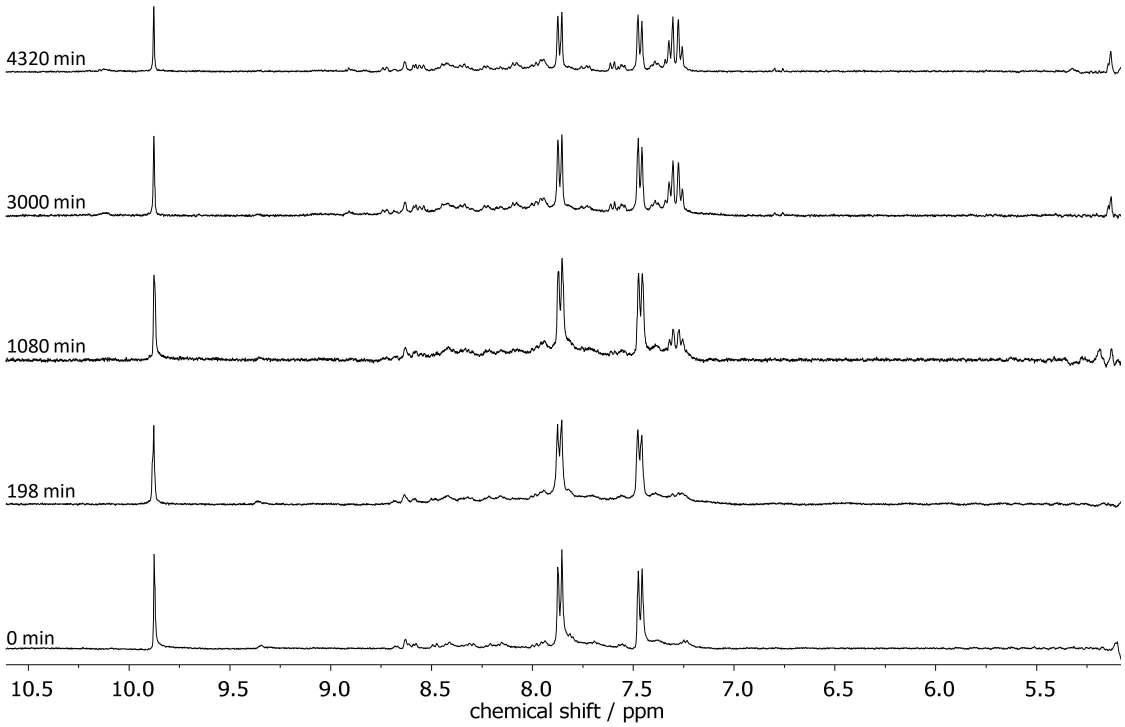

Aldol product formation:

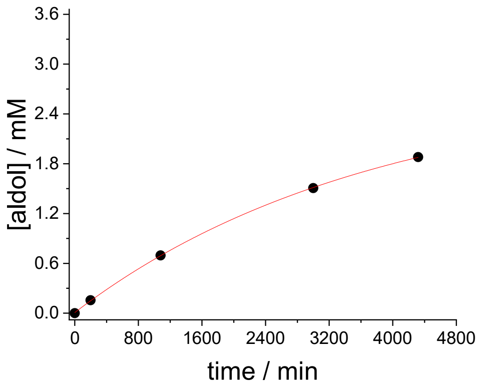

Pseudo-first-order rate constant: $\mathbf{k_{obs} = 1.7 \times 10^{-4}\ min^{-1}}$

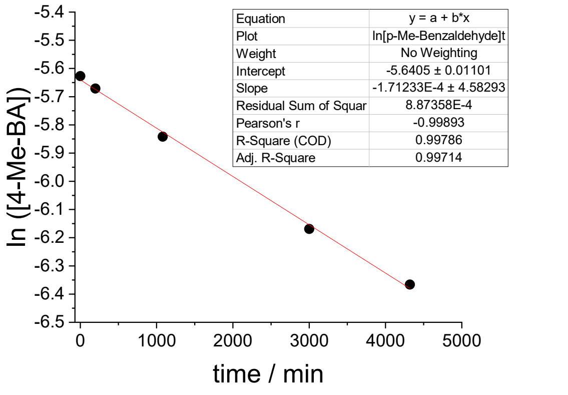

Figure S24. Partial ^{1}H NMR spectra for the aldol reaction between 4-methylbenzaldehyde and acetone. Experimental conditions: **1** (1.1 mM), 4-methylbenzaldehyde (3.6 mM), acetone (270 mM) in phosphate-buffered D_2O (45 mM at pD 8.5) at 298 K and different times.

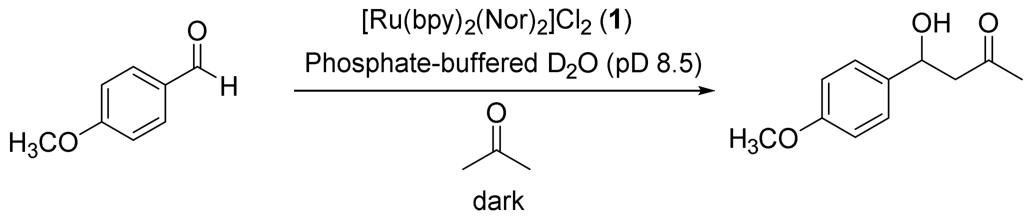

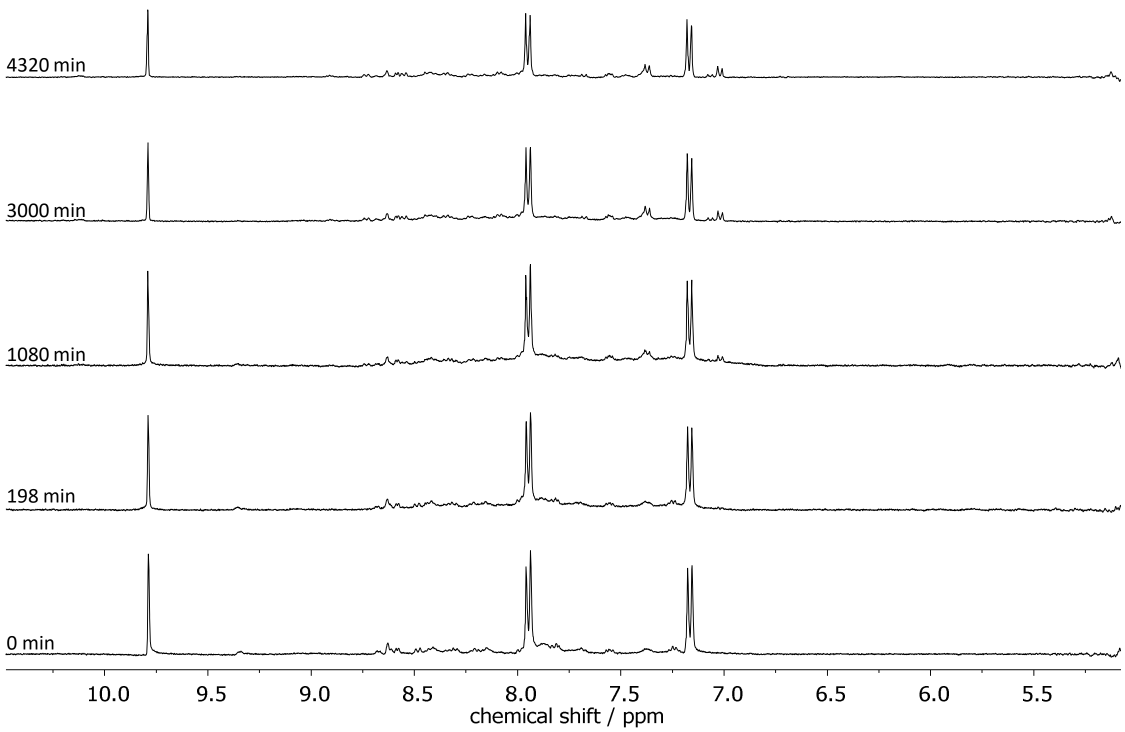

Aldol product formation:

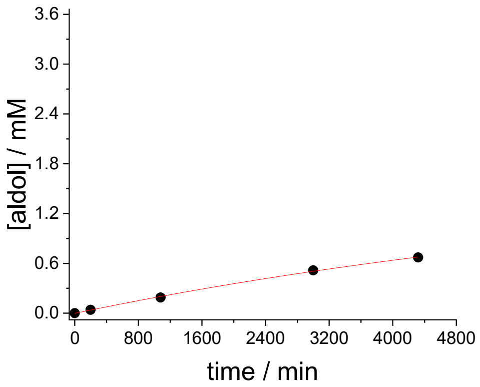

Pseudo-first-order rate constant: $\mathbf{k_{obs} = 4.9 \times 10^{-5}\ min^{-1}}$

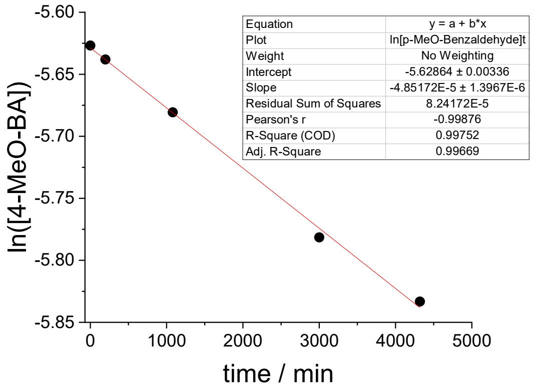

Figure S25. Partial ^{1}H NMR spectra for the aldol reaction between 4-methoxylbenzaldehyde and acetone. Experimental conditions: **1** (1.1 mM), 4-methoxybenzaldehyde (3.6 mM), acetone (270 mM) in phosphate-buffered D_2O (45 mM at pD 8.5) at 298 K and different times.

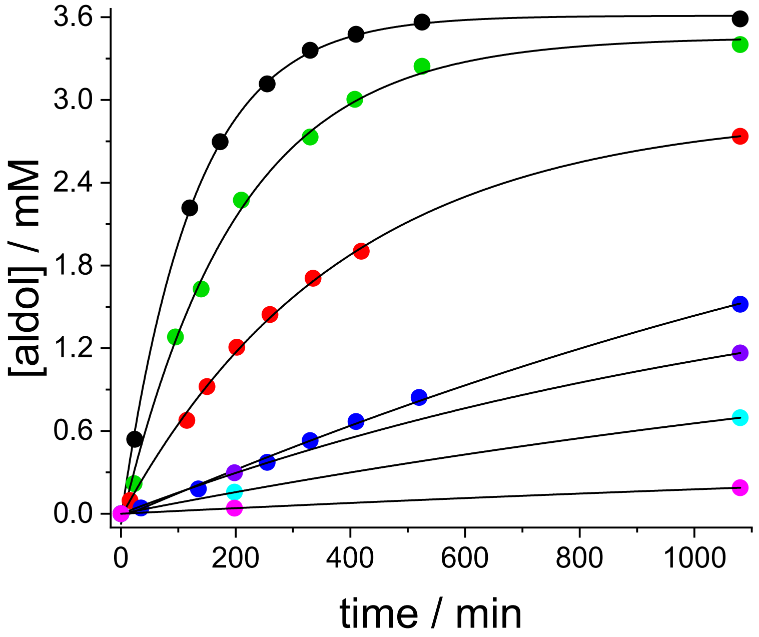

Figure S26. Comparative kinetics for different *para*-substituted benzaldehydes and parent benzaldehyde in the aldol reaction catalyzed by **1**. Colour coding by substituents: **NO_2** (black), **CN** (green), **CF_3** (red), **Cl** (blue), **H** (violet), **CH_3** (cyan), **OCH_3** (magenta).

4. Photorelease of nornicotine from 1

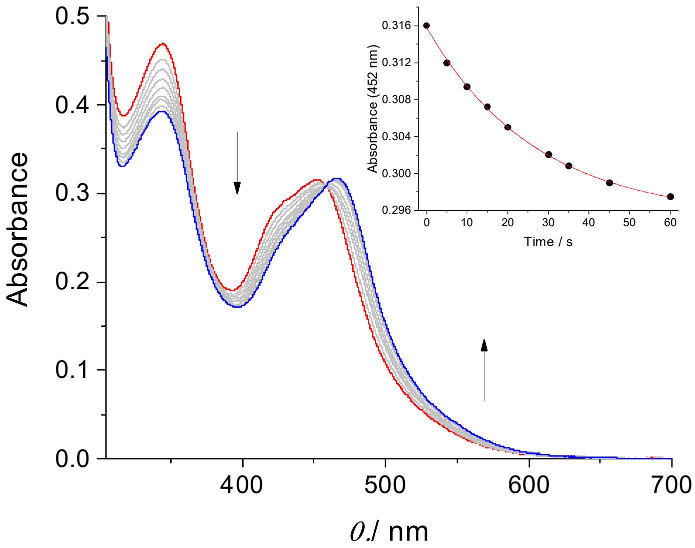

Figure S27. UV/vis absorption spectral changes on irradiation (ca. 1.8 mW cm^{-2} at > 455 nm and 298 K) of **1** (20 µM) in phosphate-buffered D_2O (pD 8.5). The blue spectrum corresponds to the endpoint of irradiation (t = 60 s). The inset show the corresponding kinetics, followed at 452 nm.

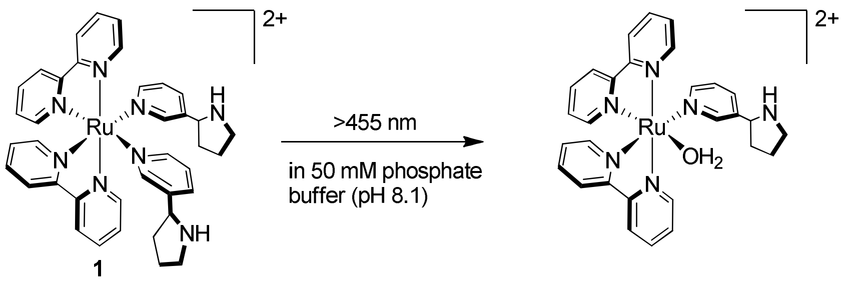

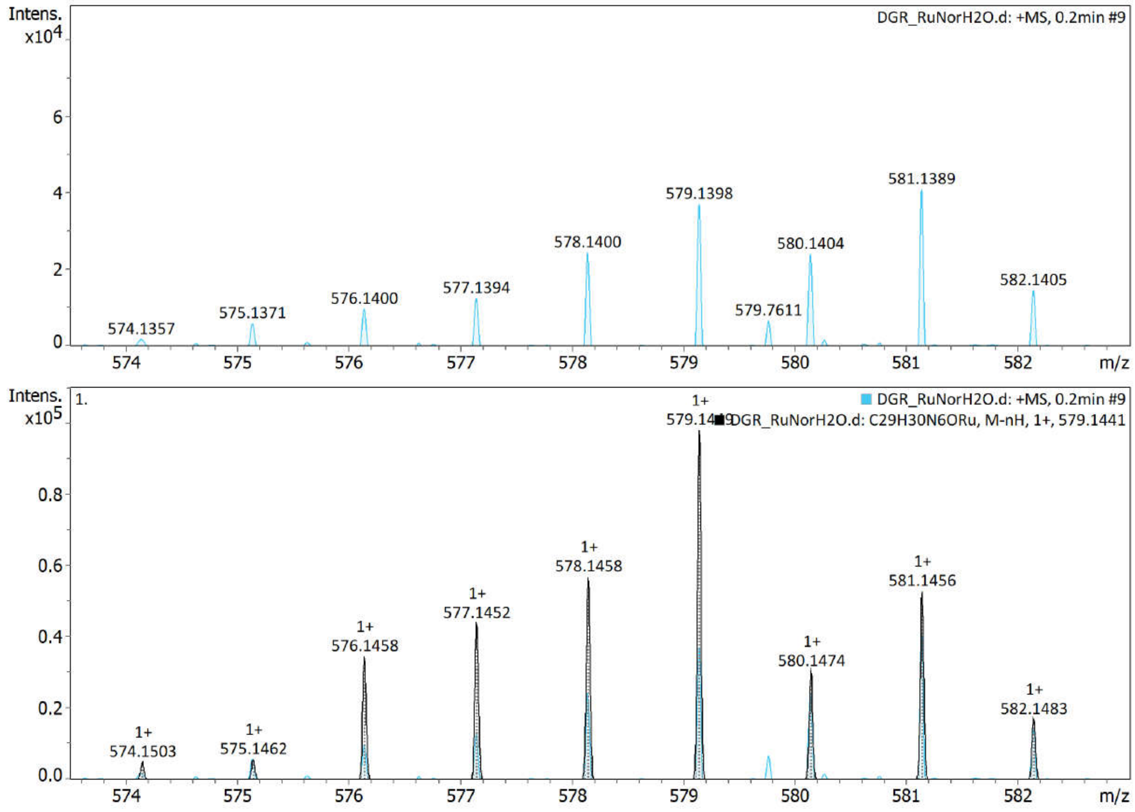

Figure S28. Top: HRMS (QTOF) spectrum of the photoproduct. Bottom: Calculated isotopic pattern (overlapped in black). Calculated for $[M-H]^+$; $C_{29}H_{30}N_6ORu$ 579.1449; found 579.1398.

5. High-resolution mass spectrometry of the Zn(II) complex with nornicotine

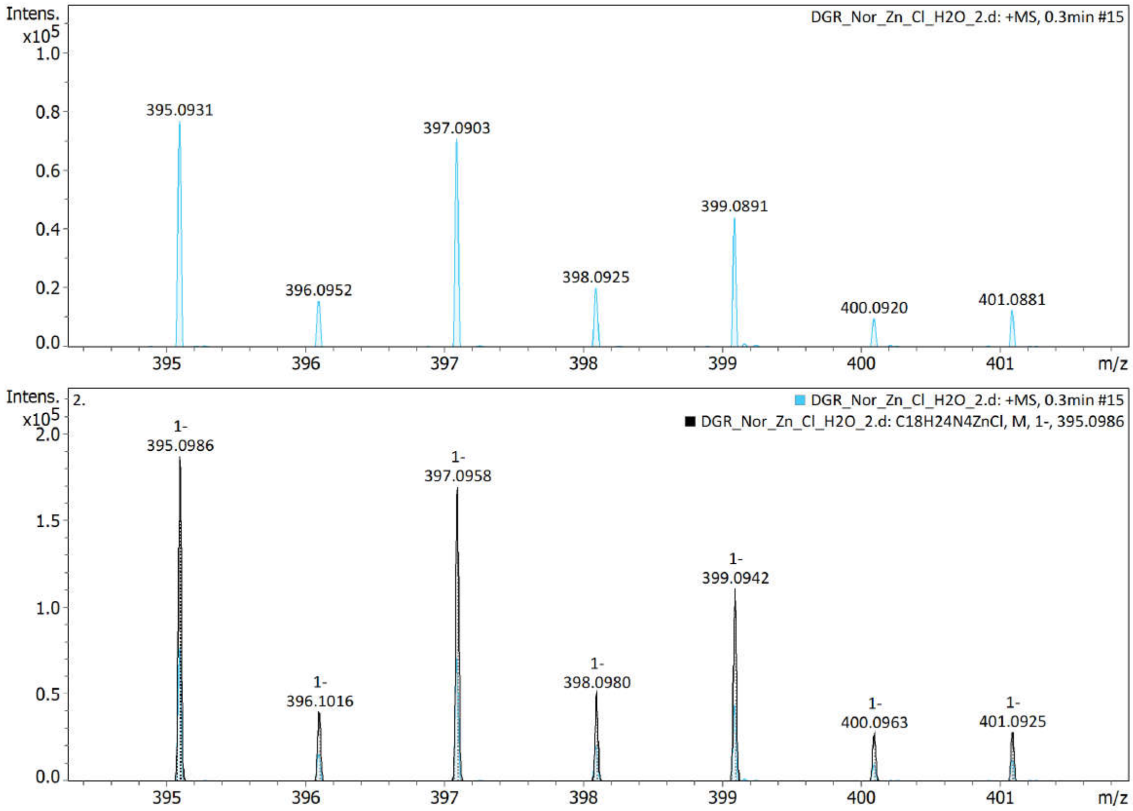

Figure S29. Top: HRMS (QTOF) spectrum of a 1:1 mixture of $ZnCl_2$ (1.2 mM) with (±)-nornicotine (1.2 mM) in water (pH 7.0). Bottom: Calculated isotopic pattern (overlapped in black). Calculated for M^+ ($[Zn(Nor)_2]Cl^+$; $C_{18}H_{24}ClN_4Zn$) 395.0986; found 395.0931.

6. High-resolution mass spectrometric evidence for enamine formation

1.2 mM **1** in presence of 300 mM acetone in phosphate-buffered D_2O (50 mM, pD 8.5), 3 hours reaction time.

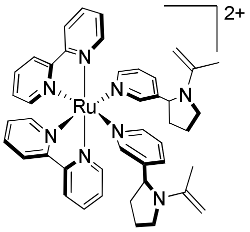

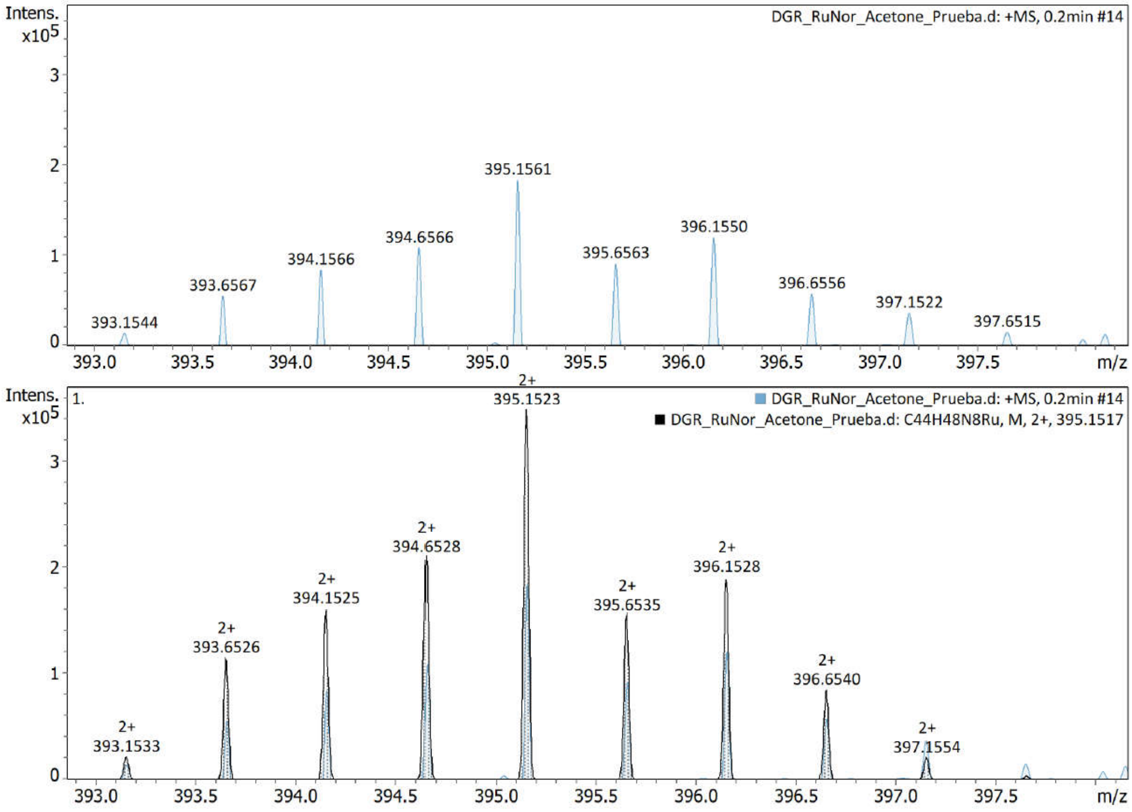

Figure S30. Top: HRMS (QTOF) spectrum. Bottom: Calculated isotopic pattern (overlapped in black). Calculated for $[M]^{2+}$; $C_{44}H_{48}N_8Ru$ 395.1523; found 395.1561. 1.2 mM **1** in presence of 300 mM acetone in phosphate-buffered D_2O (50 mM, pD 8.5), 3 hours reaction time.

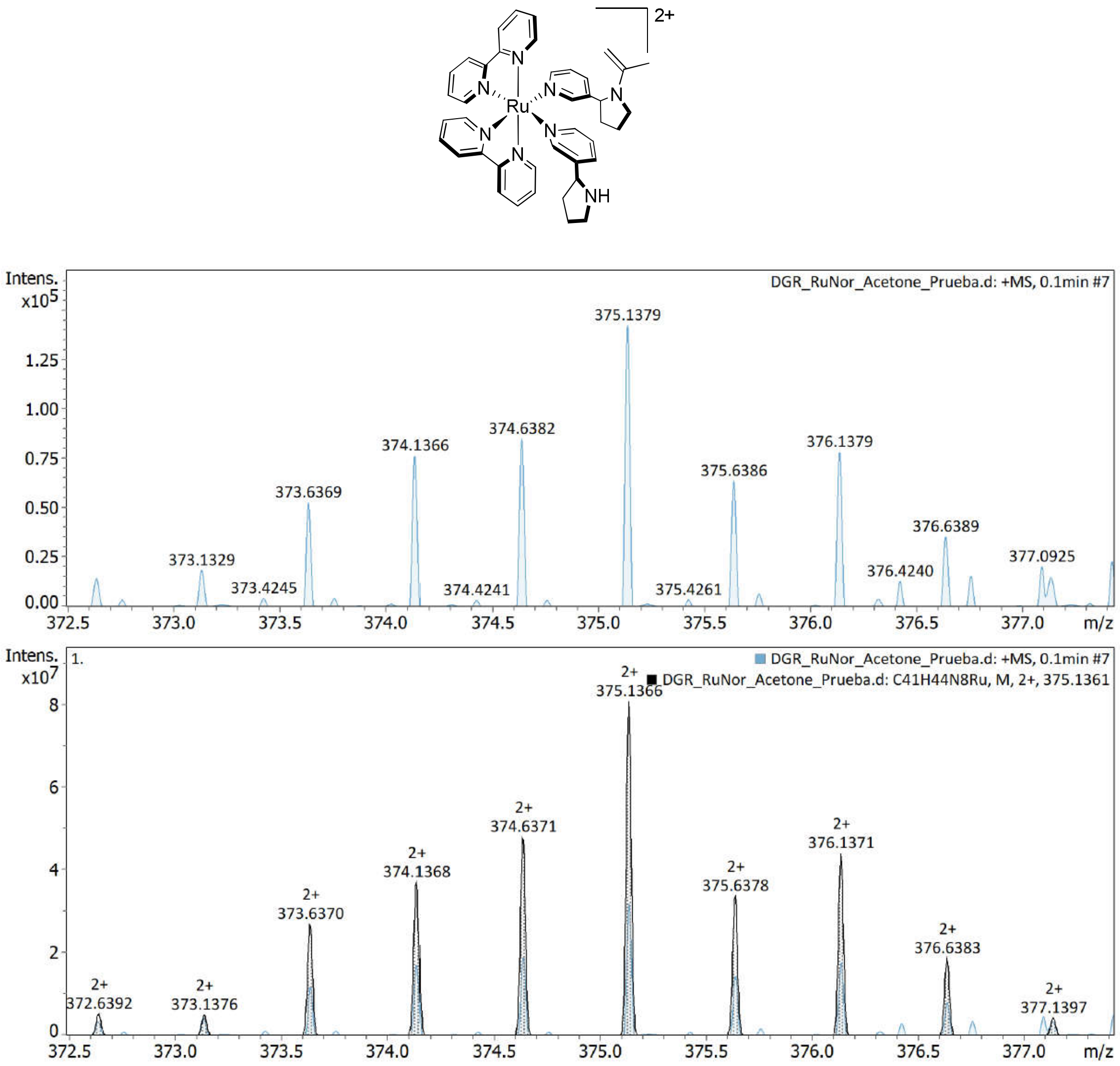

Figure S31. Top: HRMS (QTOF) spectrum. Bottom: Calculated isotopic pattern (overlapped in black). Calculated for $[M]^{2+}$; $C_{41}H_{44}N_8Ru$ 375.1366; found 375.1379.

1:1 mixture of $ZnCl_2$ (1.2 mM) with (±)-nornicotine (1.2 mM) in water (pH 7.0) in the presence of 300 mM acetone, 3 hours reaction time.

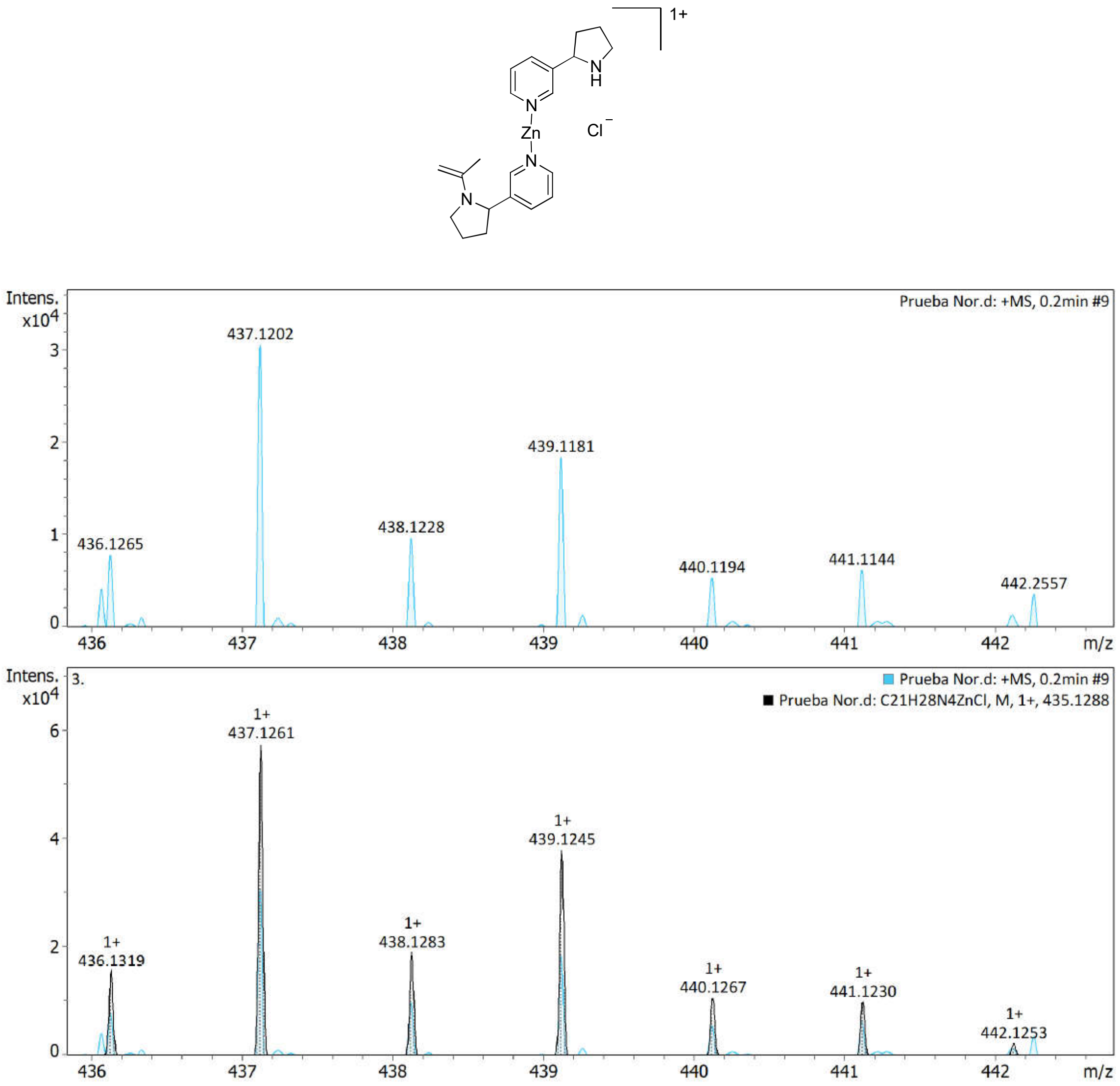

Figure S32. Top: HRMS (QTOF) spectrum. Bottom: Calculated isotopic pattern (overlapped in black). Calculated for $[M]^+$; $C_{21}H_{28}ClN_4Zn$ 437.1261; found 437.1202.
